# Machine learning of serum metabolic patterns encodes early-stage lung adenocarcinoma

**DOI:** 10.1038/s41467-020-17347-6

**Published:** 2020-07-16

**Authors:** Lin Huang, Lin Wang, Xiaomeng Hu, Sen Chen, Yunwen Tao, Haiyang Su, Jing Yang, Wei Xu, Vadanasundari Vedarethinam, Shu Wu, Bin Liu, Xinze Wan, Jiatao Lou, Qian Wang, Kun Qian

**Affiliations:** 1 State Key Laboratory for Oncogenes and Related Genes, School of Biomedical Engineering, Shanghai Jiao Tong University, 200030 Shanghai, P. R. China; 2 Department of Laboratory Medicine, Shanghai Chest Hospital, Shanghai Jiao Tong University, 200030 Shanghai, P. R. China; 3 iMS Clinic, 310052 Hangzhou, P. R. China; 4 Department of Chemistry, Southern Methodist University, 3215 Daniel Avenue, Dallas, TX 75275-0314 USA

**Keywords:** Mass spectrometry, Cancer metabolism, Tumour biomarkers, Biomedical engineering

## Abstract

Early cancer detection greatly increases the chances for successful treatment, but available diagnostics for some tumours, including lung adenocarcinoma (LA), are limited. An ideal early-stage diagnosis of LA for large-scale clinical use must address quick detection, low invasiveness, and high performance. Here, we conduct machine learning of serum metabolic patterns to detect early-stage LA. We extract direct metabolic patterns by the optimized ferric particle-assisted laser desorption/ionization mass spectrometry within 1 s using only 50 nL of serum. We define a metabolic range of 100–400 Da with 143 m/z features. We diagnose early-stage LA with sensitivity~70–90% and specificity~90–93% through the sparse regression machine learning of patterns. We identify a biomarker panel of seven metabolites and relevant pathways to distinguish early-stage LA from controls (*p* < 0.05). Our approach advances the design of metabolic analysis for early cancer detection and holds promise as an efficient test for low-cost rollout to clinics.

## Abstract (web summary)

Early diagnosis significantly improves the probability of successful cancer therapy. Here, the authors develop a technique to analyse serum metabolites and define a biomarker panel for early-stage lung adenocarcinoma diagnosis.

## Introduction

Early diagnosis improves the survival rates of many types of cancer. For lung adenocarcinoma (LA), which accounts for almost half of all lung cancers and has a mortality rate up to 80%, early diagnosis can increase the 5-year survival rate to 52% and reduce the costs of management of the disease^1^. However, conventional diagnostics using proteomic/genomic biomarkers or in vivo imaging are limited considering the detection throughput, diagnosis accuracy, analysis speed, and sampling invasiveness, particularly for early-stage LA^2,3^.

Serum analysis holds promise for early diagnosis of LA^4^ and is superior to traditional biopsy and computed tomography (CT) methods^5^, because serum analysis is non-invasive and low-cost for point-of-care testing (POCT)^6,7^ and has the desirable adaptability for universal applications. Most current serum analysis for the diagnosis of LA relies on selected genomic^8,9^ or proteomic^10^ biomarkers with limited sensitivity and specificity.

Metabolic serum analysis is more distal over genomic and proteomic approaches for precision diagnostics^11–13^, but it has rarely been reported or studied for complex diseases such as LA, due to the lack of efficient metabolite detection tools and systematically designed patient sub-groups. Changes in metabolism are associated with diverse diseases including LA^6,14^. Specifically, malignant transformations are associated with altered metabolic pathways for biosynthetic and bioenergetic processes, which depict an adjustment in blood metabolomics. Serum metabolite-guided approach has been applied to detect blood metabolic fingerprints and to identify biomarkers in various diseases, including pancreatic adenocarcinoma^15^, acute myeloid leukaemia^16^, and hepatic steatosis^17^, etc. These changes can be used for diagnostic purposes, hence the intense interest in extracting and deciphering serum metabolic information. Therefore, it is urgent to construct an advanced analytical tool for the metabolic screening of early-stage diseases, including LA.

Spectrometry methods, including nuclear magnetic resonance (NMR)^18^ and mass spectrometry (MS), particularly laser desorption/ionization (LDI) MS, enable high-throughput extraction and measurement of metabolomic information, while tandem MS allows accurate identification of metabolites^19^. However, the metabolite abundance and sample complexity affect MS analysis, and rigorous pre-treatment procedures are required for enrichment and separation of metabolites from complex bio-mixtures.

Substrates decide the efficacy of LDI MS. The tailoring of material interfaces optimizes designed interactions between molecules and substrate materials for analytical use^20,21^. For LDI MS, there have been global efforts, including ours, to engineer substrate materials^22–24^. An ideal substrate material for LDI MS-based metabolic analysis should have the following properties: (1) nanoscale surface roughness with stability for the selective LDI of metabolites^25^; (2) favourable surface charge for ion formation and conductivity for electron transfer^26^; and (3) easy preparation with low costs for mass production aimed at large-scale clinic use. The current materials being used, including noble metals^27,28^, silicon^26^, carbon^29^, metal oxides^23^, and their hybrids, only have some of these properties, so novel material-based platforms combining all of the above merits are a pressing need for the practical use of LDI MS in clinics.

A further challenge is the processing of MS big data in serum samples to obtain the necessary accuracy. Machine learning of imaging and omic information has enjoyed huge success for diagnostic use in clinics^30^. Compared with in vivo imaging and biopsy methods^31,32^ that require expensive and invasive equipment, in vitro omics diagnostic methods are advantageous, although they require big data. As one of the major tools for omic information collection, MS techniques^33,34^ (such as MasSpec Pen for cancer tissues) have afforded big data for processing and interpretation by machine learning. Notably, the selection and optimization of algorithms are required to apply machine learning in disease diagnostics.

Due to the biological significance of small metabolites (molecular weight (MW) <1000 Da) as end products of pathways and limitation performance of LDI MS in complex bio-mixtures, tackling the major problems in sample treatment, substrate materials, and data analysis for MS will lead to insights into metabolic pathways and identify effective diagnostic metabolic biomarkers. Here, we optimize the LDI MS approach to analyse a large range of metabolites (including biologically relevant metabolites) as metabolic patterns from serum samples without pretreatment by improving the substrate used. Further encoded by machine-learning algorithm, the serum metabolic patterns achieve high specificity and sensitivity diagnosis of early-stage LA and enable large-scale and low-cost rollout for use in clinics. Our approach contributes to the design of advanced metabolic analysis protocols for use in the development of precision medicine, and will lead to the development of personalized diagnostic tools for diverse diseases including but not limited to LA in the near future.

## Results

### Optimization of substrate material for selective LDI MS

**Fig. 1:**
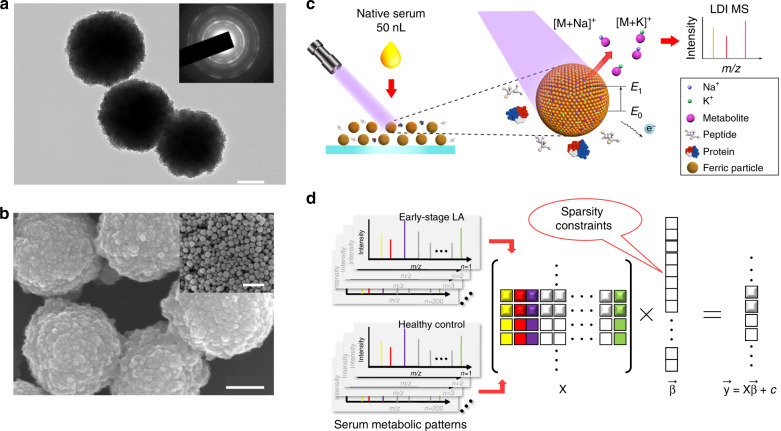
Substrate material characteristics and schematics of extraction and machine-learning workflow. **a** Transmission electron microscopy (TEM) image of ferric particles (*n* ≥ 3 randomly selected) and selected area electron diffraction (SAED) pattern (inset) showing polycrystalline structure. Scale bar = 100 nm. **b** Scanning electron microscopy (SEM) images (*n* ≥ 3 randomly selected) of ferric particles showing nanoscale surface roughness and large-scale uniformity (inset). Scale bars = 100 nm in **b** and 1 μm in the inset of **b**. **c** Schematic workflow for the extraction of serum metabolic patterns by ferric particle-assisted laser desorption/ionization mass spectrometry (LDI MS). Fifty nanolitres of native serum was consumed for direct analysis without pre-treatment procedures. Only Na^+^-adducted and K^+^-adducted metabolites can be selectively detected with the coexistence of high concentration of peptides and proteins. **d** Schematic outline for the sparse regression machine learning of serum metabolic patterns (X). The sparse regression method was used to build calculation models with sparsely constrained $\bar \beta$ towards the diagnosis of early-stage LA ($\overrightarrow {\mathbf{y}}$). Each square and its colour in X corresponded to one *m*/*z* feature and its signal intensity in serum metabolic patterns.

To enable efficient extraction of serum metabolic patterns by LDI MS, we first prepared ferric particles using a modified low-cost solvo-thermal method, yielding ~0.5 g of product from a single experiment (Fig. 1 and Supplementary Fig. 1a). Ferric particles consisted of nanocrystals (~5 nm diameter) as shown by transmission electron microscopy (TEM) (Fig. 1a). High-resolution TEM (HR-TEM) (Supplementary Fig. 1b) demonstrated the polycrystalline structure of the ferric particles (Supplementary Fig. 1b) in addition to the diffraction pattern of the particles by selected area electron diffraction (SAED, inset of Fig. 1a). By scanning electron microscopy (SEM), we observed a raspberry-like morphology of the ferric particles, which were of uniform size (~300 nm diameter, polydispersity index (PDI) of 0.155) and had a rough surface (Fig. 1b and inset), which agreed with the TEM and dynamic light scattering (DLS) results (Supplementary Fig. 1c). These particles exhibited a large surface area of 154 m^2^ g^−1^ (Supplementary Fig. 1d) validating the existence of crevices on the rough surface to selectively accommodate metabolites other than proteins, and could undergo simple and fast (~45 s) separation with a magnet due to the superparamagnetic property (Supplementary Fig. 1e). We investigated the laser absorption properties of particles and showed strong absorption in the ultraviolet–visible region of 270–1100 nm (Supplementary Fig. 1f). We concluded that these ferric particles with designer structure might be ideal as a matrix for LDI MS.

Optimizing the surface charge of substrate particles is critical for the LDI MS process of extracting serum metabolic patterns to allow ion formation and conductivity for electron transfer (Fig. 1c). We controlled the surface charge of the ferric particles during synthesis (Supplementary Fig. 2a), demonstrating that negatively charged particles with a zeta potential of –11.5 ± 2.65 mV produced by 0.4 g trisodium citrate afforded the optimized serum metabolite profile in LDI MS (Supplementary Fig. 2b) due to the enhanced formation of a positive metal ion layer on the surface to produce cation-adducted species. From 0 to 0.4 g of trisodium citrate, the metabolite signals with a signal-to-noise ratio (S/N) > 3 increased in number. Further increasing the amount of trisodium citrate resulted in no improvement in the number of metabolite signals. In addition, the ferric particles we produced had a specific band gap of <3 eV, with specific ultraviolet absorption that could be easily excited (from ground state E_0_ to excitation state E_1_) by a 355 nm laser for facile electron transfer during ionization (Fig. 1c).

We also compared LDI MS results using the conventional organic matrix (α-cyano-4-hydroxycinnamic acid, CHCA) and inorganic matrices (silica and carbon nanoparticles) together with blank controls using no matrices, showing either strong interference in low mass range or limited sensitivity/selectivity in the analysis of bio-samples to demonstrate the superiority of our approach (Supplementary Fig. 3). Specifically, as control experiments, we observed no signals by LDI MS without any matrix due to low LDI efficiency (Supplementary Fig. 3a). We obtained overwhelming background noises with few peaks from small metabolites using the organic matrix (CHCA) and carbon particles (Supplementary Fig. 3b, c) and could only recognize glucose signal using silica nanoparticles (Supplementary Fig. 3d), all of which demonstrated the advantages of ferric particles over current matrices. Notably, the rough surface of the particles offered abundant cavities for the selective and sensitive LDI of small metabolites in the presence of salts and proteins (Supplementary Fig. 4a–c), while the stable crystalline structure prevented unwanted fragmentation under laser irradiation. The features of the ferric particles that we designed promised the efficient extraction of metabolic patterns from complex fluids (e.g. serum) based on selective LDI that would enable subsequent data analysis (Fig. 1d).

There are four major aspects as rationales to select ferric particles as the substrate for our described method, including photo-thermal properties, preparation process, structural stability, and experimental cost. For photo-thermal properties, ferric particles show strong laser absorption (absorption coefficients at 355 nm as ~3.6 × 10^5^ cm^−1^) and low thermal conductivity (heat capacity as 653 J (kg K)^−1^). Thus, ferric particles can be heated to a high temperature by the laser irradiation, towards the efficient molecular desorption^35,36^. For preparation process, the solvo-thermal method required is facile to synthesize the ferric particles and the yield of ~0.5 g of product can be used to detect ~10^6^ samples for large-scale clinical use. For comparison, the preparation of various types of silicon substrates requires complicated devices and procedures, such as micro-electro-mechanical system (MEMS)^37^. For structural stability, ferric particles with stable polycrystalline structure prevented unwanted fragmentation under laser irradiation, compared to carbon nanomaterials (Supplementary Fig. 3c) that produced unavoidable carbon cluster peaks in the low MW region at high laser fluence^38,39^. For experimental cost, the ferric particles (~£0.05 g^−1^) are much cheaper, compared with noble metals (~£36.36 g^−1^ for gold), silicon (~£3.59 g^−1^), and carbon (~£0.30–43.72 g^−1^).

### Extraction of serum metabolic patterns

**Fig. 2:**
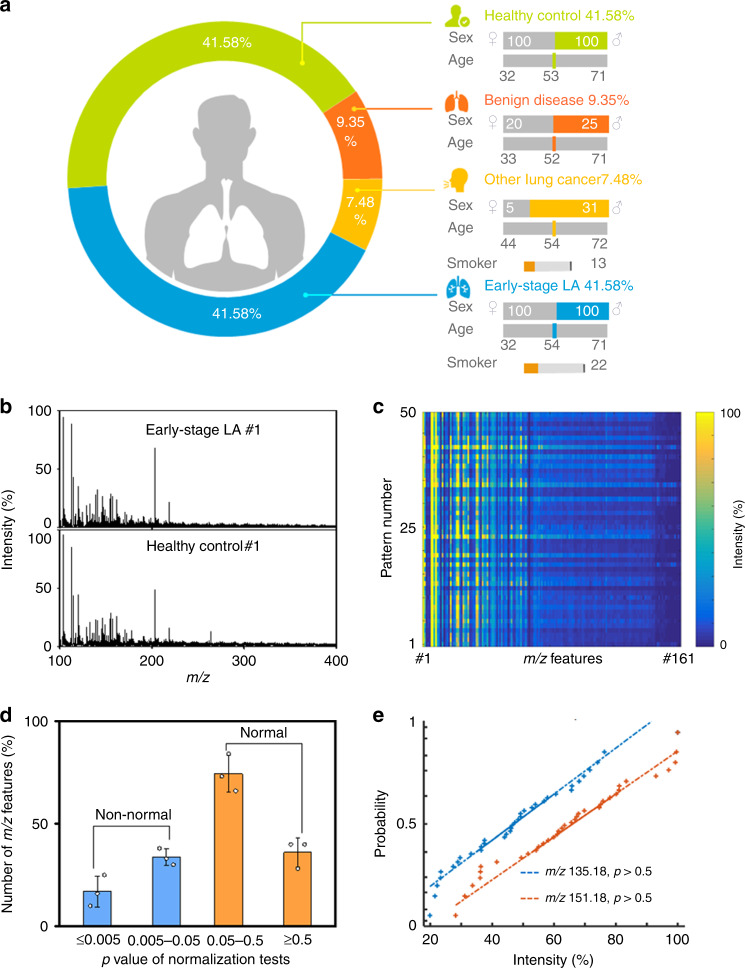
Extraction of serum metabolic patterns. **a** Demographics of 481 clinical samples. The ages of different cohorts were matched with no significant difference (*p* > 0.05). **b** Typical mass spectra (serum metabolic patterns) showing with *m*/*z* ranging from 100 to 400 obtained by optimized ferric particle-assisted LDI MS of serum samples from an early-stage LA patient and a healthy control. **c** Heat map of 50 independent metabolic patterns for one early-stage LA serum sample based on 161 *m*/*z* features from the Otsu algorithm. **d** The *p* value distribution of *m*/*z* features from normalization tests of three healthy control serum samples in parallel (50 patterns for each sample). The error bars were calculated as s.d. of three samples. Data were shown as the mean ± s.d. (*n* = 3). The *m*/*z* features with *p* > 0.05 and *p* < 0.05 represent normal and non-normal distributions, respectively (two-sided Lilliefors (Kolmogorov–Smirnov) test with no adjustment made for multiple comparisons). **e** Probability of a normal distribution of *m*/*z* features at 135.18 (blue) and 151.18 (orange) for 50 patterns of one serum sample from healthy control, both with *p* > 0.5 (*n* = 50 independent experiments, two-sided Lilliefors (Kolmogorov–Smirnov) test with no adjustment made for multiple comparisons). Dotted lines are the reference lines for normal distribution. Source data are provided as a Source Data file.

Having optimized the substrate, we tested the ability of ferric particle-assisted LDI MS, to extract serum metabolic patterns from patients. A total of 481 serum samples from 200 patients with early-stage LA, 200 healthy controls, 36 patients with other lung cancer, and 45 with benign lung diseases were included. The blood was drawn at initial diagnosis, without surgery or anaesthesia. The blood collection for each subject enroled in this project was following the same protocol. We also included power analysis (a universal method to derive the optimal sample size by estimating statistical power in a hypothesis test) on a dataset from a pilot study of 12 samples (6/6, LA/control) to compute the minimum sample number required for the meaningful machine learning (Supplementary Fig. 5). Based on the power analysis result, the minimum number of samples was 200 (100/100, LA/control) with predicted power ~0.8 at a false discovery rate (FDR) of 0.1, which can be a sufficient confidence level to conclude the statistical meaningful results according to previous refs. ^40,41^. All patients were diagnosed by pathologists and the tumours were staged according to the international standards for tumour, node, and metastasis (TNM) staging of lung cancer^1^. No significant age difference was observed among groups (*F* = 0.088, *p* = 0.767, by one-way analysis of variance (ANOVA); Fig. 2a and Supplementary Table 1).

We yielded direct mass spectra from all 481 serum samples by ferric particle-assisted LDI MS without enrichment or separation. We firstly extracted 810 peaks from the raw MS data, by searching the localized highest intensity. We further identified 161 *m*/*z* features out of 810 peaks for the serum of both early-stage LA patients and healthy controls (Supplementary Fig. 6) based on the Otsu algorithm^42,43^, by estimating the threshold and deciding the background noise on the maximum interclass variance and excluding random background peaks from 810 peaks according to the threshold. In particular, 89% (143 *m*/*z* features) out of 161 *m*/*z* features were located in the low mass range (100–400 *m*/*z*; Fig. 2b). These 161 *m*/*z* features were considered as the final MS output (metabolic pattern) for the disease classifier.

We collected 50 independent metabolic patterns for one early-stage LA serum sample and plotted the heat map, showing that the metabolite signals were distributed vertically and uniformly in the given *m*/*z* range (Fig. 2c). Notably, 110 ± 3 *m*/*z* features were normally distributed (*p* > 0.05, Fig. 2d) at 5% significance level for three control serum samples (each with 50 independent patterns), validating the reproducibility of the metabolic pattern extraction. For instance, we showed that 50 patterns of one serum sample had a normal distribution, with peaks at *m*/*z* values of 135.18 and 151.18, both with *p* > 0.5 and close to two reference lines for normal distribution (Fig. 2e). We used the cosine correlation algorithm to investigate the spectra similarity within one group, which had been widely applied in previous literatures^44^. Typically, one spectrum was randomly selected from each group and fixed as the reference spectrum for spectra similarity analysis. As a result, we showed the frequency distribution of the similarity scores for each group (both LA and controls) in Supplementary Fig. 7. Notably, the frequency of spectral similarity scores >0.9 reached 94% and 80%, for LA and controls, respectively. The above results indicated the reliability and potency of the serum metabolic patterns obtained with ferric particle-assisted LDI MS for diagnostic applications.

Notably, prior efforts need lengthy pre-treatment procedures (~hours) and large volumes of serum (50 μL at least), to address sample complexity and metabolite abundance, respectively^19^. For comparison, our approach offers enhanced analytical speed (~seconds) and reduced sample consumption (500 nL) by ~2–3 orders of magnitude. Importantly, we found that quantitation of glucose, histamine, and mannitol using our approach afforded consistency with the standard liquid chromatography electrospray ionization (LC ESI) MS method, with the coefficient of determination (*R*) of 0.88–0.99 (Supplementary Fig. 8 and Supplementary Table 2). Our success relied on the selective LDI of small metabolites by ferric particles to produce signals in the low-mass range (*m*/*z* < 400), particularly in the presence of serum proteins and salts (Supplementary Fig. 4a, b). Further considering the high reproducibility (Fig. 2d, e) of the MS data, sensitivity (Supplementary Fig. 4c and Supplementary Table 3) of pattern extraction, and large-scale synthesis of material (Supplementary Fig. 1a) for the high-throughput screening of 161 *m*/*z* features (Fig. 2c) in serum, we next approached the major obstacles to metabolic analysis for massive clinic use.

### Diagnosis of early-stage LA by machine learning

**Fig. 3:**
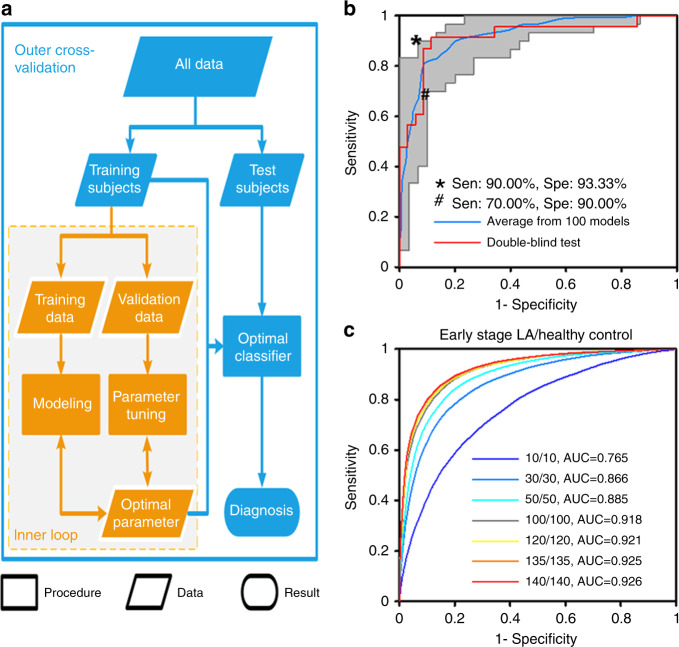
Diagnosis of early-stage LA by machine learning. **a** Schematic workflow for the construction of classification models, including an inner loop (machine-learning stage, orange) to tune the hyperparameters for the optimal classifier and outer cross-validation (classifier building stage, blue) to evaluate the discriminant performance. **b** Receiver operating characteristic (ROC) curves for the classifier designed to distinguish between early-stage LA patients and healthy controls. The colours of ROC curves—blue represented the ROC curve obtained by averaging 20 rounds of five-fold nested cross-validations (100 models in total) with a mean AUC of 0.921 (95% confidence interval (CI): 0.891–0.953), and the optimized number of training subjects was 240 (120/120, LA/control); red represented the ROC curve obtained from double-blind test (23/35, LA/control), showed AUC of 0.915 with diagnostic sensitivity of 88.57% and specificity of 91.30%; the grey area indicated the specificity/sensitivity of all independent ROC curves from 100 models, showing the diagnostic performance of the best (asterisk) and worst (hash mark) classifiers. **c** Averaged ROC curves with AUC to optimize the number of training subjects, analyzing from 20 (10/10, LA/control) to 280 (140/140, LA/control). Source data are provided as a Source Data file.

To optimize the hyperparameters for the optimal classifier and evaluate the diagnostic performance of our ferric particle-assisted LDI MS approach, we performed machine learning of serum metabolic patterns (**X**) for the diagnosis (${\vec{\mathbf{y}}}$) of early-stage LA (Fig. 3a). There were two major components to our evaluation—the inner-loop for hyperparameter optimizing stage and outer-cross validation for classifier building stage—based on the sparse regression method to build calculation models with sparsely constrained $\vec \beta$, involving only a subset of the variables/predictors (Fig. 1d, see Online methods for details). We tuned hyperparameters through a nested cross-validation approach (five-fold both for the inner-loop and outer-cross validation, repeated 20 rounds, 100 models in total) to optimize the model parameters (*λ*_1_ and *λ*_2_). The training subjects were internally and randomly split into five folds for the inner-loop, to identify optimized hyperparameters on training samples in the nested cross-validation. And the case:control ratio (1:1) for the inner-loop maintained the same in each internal split, based on the five-fold cross-validation. Specifically, the discriminant performance of the classifier built from the nested cross-validation reached an average sensitivity of 90% and an average specificity of 93% (averaged from 100 models in total), based on the optimized model with wavelength *λ*_1_ = 0.035 and *λ*_2_ = 0.024 in Fig. 3b. Importantly, we recruited a new cohort (Supplementary Table 4) from Shanghai Chest Hospital, with an independent set of 58 samples (23/35, early-stage LA/healthy controls) as the double-blind test. Notably, the double-blind test cohort was independent from the training and test subjects in classifier building stage and blinding to the as-built classifier. The situations for blood drawn were the same for all sample sets. We obtained the area under the curve (AUC) of 0.915 (red line, Fig. 3b), with diagnostic sensitivity of 88.57% and specificity of 91.30%, consistent with the previous results in the spectra and algorithm development.

By adjusting the number of training subjects from 20 to 280, we obtained an increasing AUC with enhanced performance (Fig. 3c). We identified a minimum number of samples to potentially apply meaningful machine learning, by varying training sample number from 20 to 280 (Fig. 3c), while the testing set size was also varied from 20 to 280 for the nonoverlapping sample splitting in training number optimization. The minimum number of training samples was 200 (100/100, LA/control), with AUC > 0.9 for machine learning. We identified the optimized number of training subjects as 240 (120/120, LA/control), showing limited improvement with further increases in the number of training subjects. Notably, the models were robust without overfitting, due to the nonoverlapping sample splitting in training number optimization and the consistent performance with double-blind test. For the nonoverlapping sample splitting, the whole samples were split into nonoverlapping training and test set by cross-validation, which is universally employed to avoid information leakage during each training step and prevent overfitting^45,46^. For the double-blind test, we demonstrated the discriminant performance (AUC of 0.915) by double-blind test in diagnosis was consistent with the results (AUC of 0.921) by cross-validation in classifier building. Notably, the double-blind test cohort was independently enrolled, decreasing the risk of model overfitting and refusing overly optimistic results. The consistency between double-blind test and cross-validation further guaranteed a robust model without overfitting, according to previous reports^46,47^. Recently reported proteomic and genomic approaches (with AUC of ~0.6–0.9) require time-consuming (~hours) reactions (e.g. immunoassay and polymerase chain reaction) that are not ideal for routine clinical use^4,48^. For comparison, our metabolic approach provided desirable analytical performance (speed of ~seconds) and diagnostic performance (AUC of ~0.9) for early-stage LA detection in serum, demonstrating that computer-aided diagnosis based on serum metabolic patterns detects early-stage LA.

### Construction of the metabolic biomarker panel

**Fig. 4:**
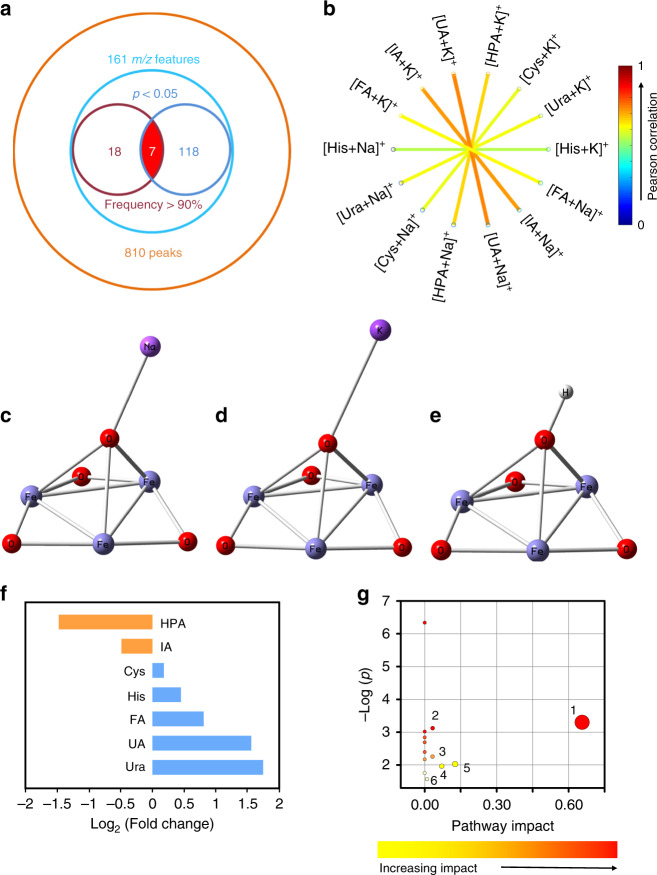
Construction of metabolic biomarker panel. **a** Venn diagram of 161 *m*/*z* features from 810 metabolite peaks in serum, seven of which were selected as potential biomarkers with both model selection frequency >90% and *p* < 0.05 (<400 Da). **b** Correlation network plot elucidating strong Pearson correlation (>0.5) between Na^+^-adducted and K^+^-adducted signals (along diagonal line) for all seven selected metabolites in serum. Binding affinity of cations on the exposed surface [1,1,1] of ferric particles. Density functional theory (DFT) calculation results of **c** [ferric particles+Na^+^], **d** [ferric particles+K^+^], and **e** [ferric particles+H^+^] system with an anionic cluster model in the minimum-energy structure. **f** Fold change of five up-regulated metabolites (blue) and two down-regulated metabolites (orange) in early-stage LA patients compared with healthy controls. **g** Potential pathways differentially regulated in early-stage LA patients and healthy controls. The seven selected metabolites were tested to identify altered pathways. The colour and size of each circle were correlated to the *p* value and pathway impact value. A total of six pathways were differentially regulated: (1) fatty acid metabolism, (2) sulfur metabolism, (3) histidine metabolism, (4) cysteine and methionine metabolism, (5) pyrimidine metabolism, and (6) purine metabolism. Pathways with impact values >0 were considered to be differentially altered between early-stage LA patients and healthy controls. Source data are provided as a Source Data file.

We further set out to find metabolic biomarkers (also as potential therapeutic targets) in patterns to characterize relevant pathways. We identified a biomarker panel of seven metabolites (<400 Da) based on accurate mass measurement (for both Na^+^- and K^+^-adducted signals) and tandem MS (Fig. 4a, Supplementary Figs. 9–15, Supplementary Table 5), accounting for an AUC of 0.894 (Supplementary Fig. 16a). The panel consisted of: uracil (Ura), histamine (His), cysteine (Cys), 3-hydroxypicolinic acid (HPA), uric acid (UA), indoleacrylic acid (IA), and fatty acid (FA) (18:2). Notably, a strong Pearson correlation between Na^+^-adducted and K^+^-adducted signals (>0.5) for the seven metabolites validated the presence and role of these metabolites as biomarkers (Fig. 4b, Supplementary Fig. 17). Specifically, we computed the odds ratios of the metabolic biomarkers in a logistic regression model (referred to the basic model) and adjusted for age and sex, according to previous reports^49^. As a result, age and sex were not significant covariates for any metabolic biomarker and thus the seven metabolites retained significant odds ratios (≠ 1) when adjusted for age and sex (Supplementary Table 6). The localized mass spectra and scatter plots for serum metabolic patterns showed significant differences (*p* < 0.05, Supplementary Figs. 18 and 19) between early-stage LA and healthy controls for each biomarker.

There are two aspects regarding the breadth of metabolites, including both chemical (molecular structure) and physical (molecular size) properties. For molecular structure, metabolites containing polar functional groups (like hydroxyl group) can be cationized on the surface of ferric particles, through the dipole–dipole interaction^50,51^. Therefore, our approach exploits an ability to produce cation (Na^+^, K^+^)-adducted metabolite species for polar compounds (e.g. amino acids, polyamines, carbohydrates, organic acids, nucleosides, etc.). For molecular size, only small metabolites (MW < 1000 Da) can be selectively accommodated and trapped by the nano-crevices (~nm) of ferric particles, due to the size-exclusive effect as demonstrated in literatures^22,52^. Therefore, the surrounding alkali metal ions in the nano-crevices may facilitate efficient LDI of small metabolites typically with MW < 1000 Da. Notably, we did not observe H^+^-adducts by using ferric particle-assisted LDI MS, which was validated by the standard molecule detection (Supplementary Fig. 20) and consistent with previous reports^35,53^. Importantly, to further investigate the ion adduction process and characterize the competing adduction effect regarding H^+^/Na^+^/K^+^, we performed quantum simulation with density functional theory (DFT) calculation to the exposed surface [1,1,1] of ferric particles (Supplementary Fig. 21). The binding affinity of H^+^ is −13.6 eV (Fig. 4c) on the surface of ferric particles, much higher than those of Na^+^ (−4.7 eV, Fig. 4d) and K^+^ (−4.0 eV, Fig. 4e), hindering the cation transfer to analytes and coupled cationization.

Notably, we found that uracil (increases of 3.36-fold) and UA (increases of 2.95-fold) were the most highly altered species with over expression, while HPA was the most highly altered specie with down expression (Fig. 4f). Principle component analysis (PCA) of these seven metabolites (Supplementary Fig. 16b) displayed enhanced clustering, compared with that of all 161 *m*/*z* features (Supplementary Fig. 16c) between early-stage LA and healthy controls. Single one of these biomarkers cannot be very useful in discriminating disease from control samples. Only poor AUC (<0.7) can be acquired by univariate receiver operating characteristic (ROC) curve analysis for single one of these biomarkers (Supplementary Table 5). Importantly, the combination of seven biomarkers together accounted for an enhanced AUC of 0.894 by multivariate ROC curve analysis, in differentiating early-stage LA from healthy controls (Supplementary Fig. 16a), compared to the poor diagnostic performance by single one of these biomarkers (AUC < 0.7). Therefore, we concluded that the panel of seven biomarkers was useful in discriminating disease from control samples. The success can be attributed to that multivariate analysis by combined biomarkers is superior to univariate analysis by one single biomarker, which had been well established and recognized in literatures^4,54^. The construction of the biomarker panel facilitated the simple analysis and large-scale use of our approach in clinics.

We also performed further data analysis to demonstrate the metabolic differences and similarities, among early-stage LA and other lung cancers/benign diseases (Supplementary Table 1). For metabolic differences, we identified another two new panels of metabolites based on the metabolic patterns, to differentiate early-stage LA from other lung cancers/benign diseases. Notably, the two panels showed superior diagnostic performance, due to the metabolic differences related to disease phenotypes (Supplementary Fig. 22, Supplementary Tables 7 and 8). For metabolic similarities, we identified the overlapping metabolites that were differentially expressed, among early-stage LA and other lung cancers/benign diseases. Specifically, in the differentiation of early-stage LA and other lung cancers from healthy controls, we observed that Ura and IA were the overlapping metabolites. In parallel, in the differentiation of early-stage LA and other lung diseases from healthy controls, we observed that IA was the overlapping metabolite. Due to the pathological process of lung diseases and altered metabolic pathways, the metabolic similarities reflected the systematic response to diseases.

In-silico interrogation of potentially altered metabolic pathways (Fig. 4g, Supplementary Table 9) were analysed by the pathway topology analysis in MetaboAnalyst (http://www.metaboanalyst.ca/), displaying the major metabolic contributions from nucleotides (Ura and UA), FA, organic acids (Cys, HPA, and IA), and active amine (His). Specifically, the differential expression of Ura and UA (the nucleotide metabolism intermediate metabolites) reflected metabolic adaptation to the increased transcriptional activity and differential regulation of purine and pyrimidine metabolism due to cancer cell proliferation^11,18^. The abnormal expression of FA fit with the current theory that FA degradation is reduced in tumour cells^12,34^, which was the pathway with the most significant impact (0.656). Among the organic acids correlated with protein and energy metabolism disorders, the changes in Cys, HPA, and IA suggested differential regulation of cysteine and methionine metabolisms, and sulfur metabolism caused by the greatly increased biosynthesis of proteins and abnormal activation of degradation enzymes during tumour growth^12,55^. Finally, active amine (His) is involved in allergy and inflammation, which are involved in the cancer initiation process^56,57^. Moreover, we found six metabolic pathways were shared both in early-stage LA and other lung cancers, including (1) beta-alanine metabolism, (2) pyrimidine metabolism, (3) pantothenate and CoA biosynthesis, (4) glycine, serine, and threonine metabolism, (5) taurine and hypotaurine metabolism, and (6) histidine metabolism (Supplementary Fig. 23a). Similarly, we found (1) histidine metabolism and (2) pyrimidine metabolism were shared both in early-stage LA and benign lung diseases (Supplementary Fig. 23b). Together, we concluded that the commonly altered metabolisms were observed in lung diseases, also as demonstrated in literatures^58,59^.

Pathway topology analysis has been widely applied in biomedical research and depends on the metabolite importance and metabolite number. For metabolite importance, the importance of one compound is estimated by its centrality measure (node or edge), in a given metabolic network according to literatures^40,60^. Compared to metabolites as edges that have little impact on pathway topology analysis, metabolites as nodes (*n* = 1 or *n* = 2 metabolites) have a significant impact on pathway topology analysis. For metabolite number, low metabolite number (*n* = 1 or *n* = 2 metabolites) can be used, since the total number of metabolites varies in different metabolic networks according to literatures^61,62^. Importantly, given the criterion that pathway impact is >0 and −log(*p*) > 1, the altered pathways analysis can be driven by *n* = 1 or *n* = 2 metabolites. Typically, in FA metabolism, there are 15 metabolites in total. Among the 15 metabolites, FA (18:2) functions as a node, showing the highest importance of 0.66 and −log(*p*) of 3.30 (Supplementary Fig. 24). In pyrimidine metabolism, uracil displays importance of 0.07 and −log(*p*) of 1.96 as a node, higher than 89.74% of metabolites in the pathway. Similarly, UA displays importance of 0.009 and −log(*p*) of 1.57 as a node in purine metabolism. The criterion can be due to the high metabolite importance and/or low metabolite number in the specific pathways (e.g. FA and pyrimidine metabolism), which is universally applied in previous literatures^63,64^.

## Discussion

As a limitation of this work, the mass spectrometer system is required to detect the serum metabolic pattern, which can be subject to instrumentation in reducing its size for real case POCT. Also, we acknowledge that a certain number of pre-defined samples would be needed, as with any technology that relies on machine learning and statistical modelling of data sets, to obtain the optimized classifiers for diagnosis. Finally, the performance and outreach of this work can be strengthened, using a combination of multi-modal data from individuals in clinical study.

In summary, we extracted serum metabolic patterns using a ferric particle-assisted LDI MS approach and deciphered these patterns with a sparse regression model of machine learning for the differential diagnosis of early-stage LA. This work contributes to the design of advanced metabolic analysis protocols that will facilitate precision medicine and lead to the development of personalized diagnostic tools based on seven biomarkers for diverse diseases in the near future. Our approach may have an impact on metabolic analysis, similar to that of polymerase chain reaction on genetic analysis.

## Methods

### Chemicals and reagents

Ferric chloride (purity > 97%), trisodium citrate (purity > 99%), ethylene glycol, sodium acetate (purity > 99%), tetraethyl orthosilicate (TEOS, purity >96%), absolute ethanol (EtOH), trifluoroacetic acid (TFA, purity >99%), and ammonium hydroxide (purity > 10–35%) were purchased from Sinopharm Chemical Reagent Beijing Co. Ltd. (Beijing, China). Resorcinol (purity > 99%) was purchased from J&K China Chemical Ltd. (Shanghai, China). Albumin from bovine serum (BSA, purity >98%), CHCA (purity >99%), acetonitrile (ACN, purity >99%), standards including cysteine (purity > 99%), UA (purity > 99%), d-glucose (purity > 99.5%), sucrose (purity > 99%), d-mannitol (purity > 99%), l-leucine (purity > 98%), l-cellobiose (purity > 99.5%), l-lysine (purity > 98%), valine (purity > 99%), dl-phenylalanine (purity > 99%), and arginine (purity > 99%) were purchased from Sigma, USA. Formaldehyde solution (CH_2_O, purity >36.0%) and standards including histamine (purity > 99%), uracil (purity > 99%), HPA (purity > 99%), IA (purity > 99%), and FA (18:2) (purity > 99%) were purchased from Shanghai Aladdin Reagent Co. Ltd. (Shanghai, China). All aqueous solutions were prepared using deionized water (18.2 MΩ cm, Milli-Q, Millipore, GmbH).

### Synthesis of substrate materials

The ferric particles were prepared using a modified solvo-thermal method, that can be used for large-scale manufacturing at low cost. Briefly, ferric chloride was first dissolved in ethylene glycol solution. Trisodium citrate (weights from 0 to 0.8 g) was then added to tune the surface charge of the products. Then, 1.8 g of sodium acetate was added, and the mixture was sonicated at room temperature for 30 min. The reaction mixture was transferred to a Teflon-lined stainless-steel autoclave (capacity 50 mL) and held at 200 °C for 10 h for the formation of ferric particles. The final product was washed with ethanol and deionized water and dried at 60 °C before use.

To prepare substrate materials as controls, silica, and carbon particles were synthesized. For silica particles, a classic Stöber method was used to synthesize the monodispersed silica nanospheres^22,65^. Typically, 2 mL of ammonium hydroxide was added into the mixture of 53 mL of ethanol and 2.33 mL of deionized water under vigorous stirring for 10 min. Then 3 mL of TEOS was added into the above-mixed solution and stirred for 6 h to obtain the final silica nanoparticles. The resulting products were washed with 50 mL of ethanol and deionized water and centrifuged at 10,000×*g* for 10 min and dried at 60 °C before use. For carbon particles, polymer particles were formed with resorcinol/formaldehyde in a mixture of EtOH and aqueous ammonia, followed by carbonization. Typically, 0.2 mL of ammonium hydroxide was added to a mixture of 20 mL deionized water and 8 mL EtOH under magnetic stirring at room temperature for 1 h. Then, 0.1 g resorcinol and 0.14 g formaldehyde were introduced and stirred at room temperature for 24 h. The above solution was transferred to a Teflon-lined stainless-steel autoclave and heated for 24 h at 100 °C for the final product. The polymer particles were carbonized at 550 °C in nitrogen atmosphere.

### Characterization methods

TEM images, HR-TEM images, and SAED patterns were collected by depositing 10 μL of material suspension onto a copper grid using a JEM-2100F instrument (JEOL, Japan). SEM images were recorded on an S-4800 (Hitachi, Japan), where a drop of material suspension was placed on aluminium foil. The optical absorption spectrum of the materials was obtained on a UV1900 spectrophotometer (AuCy, China) at room temperature. The magnetic hysteresis loop was measured by a vibrating sample magnetometer (Quantum Design, USA) at 300 K. Nitrogen adsorption isotherm was obtained on an ASAP 2020M (Micromeritics, USA), and the sample was degassed in vacuum before testing. Zeta potential and DLS size measurements were performed on a Nano-ZS90 instrument (Malvern, Worcestershire, UK) in water at 25 °C.

For DFT calculation, the ferric particles were simulated with an anionic cluster model (exposed surface as [1,1,1]) reported in literature. The geometry optimization was carried out in ORCA 4.1.1 package^66,67^. The BP86 density functional with def2TZVP basis set was employed.

### MS data acquisition

For LDI MS using ferric, silica, and carbon particles, the particles were dispersed in water at a concentration of 1 mg mL^−1^. CHCA was dissolved in 0.1% TFA buffer (water/ACN, 50/50, v/v) at a concentration of 4 mg mL^−1^. Then, 500 nL of matrix slurry was mixed with 50–500 nL of analyte solution (either standards listed in chemicals and reagents part or serum samples) on the plate and dried for LDI MS analysis. The protein mixtures were prepared using established methods. Mass spectra were collected in the reflection mode employing delayed extraction on a 5800 Proteomics Analyzer (Applied Biosystems, Framingham, MA, USA) with a Nd:YAG laser wavelength of 355 nm, a repetition rate of 200 Hz and an acceleration voltage of 20 kV. The delay time for this experiment was optimized to 250 ns. The MS data can be visualized in DataExplorer (Version 4.5). Only MS signals with a signal-to-noise ratio over 3 were used for the identification of molecules, and mass calibration was conducted using standard molecules for the accurate mass measurement (±0.05 Da) of both Na^+^-adducted and K^+^-adducted signals. Tandem MS (MS/MS) was performed for selected *m*/*z* features in both standards and serum, with collision-induced dissociation (CID) off and a full-width-half-maximum (FHWM) of 500. No smoothing procedures were applied, and all spectra were directly used for analysis.

For LC ESI MS method, 28 mixed samples were prepared containing different content of glucose, histamine, and mannitol (see details in Supplementary Table 2). The isotopes of glucose and mannitol were introduced as the internal standard for quantification use both in LDI MS and in LC ESI MS. The isotopes were dissolved in water with concentrations of 200 ng μL^−1^ and mixed with analyte solutions. For LDI MS analysis, after dropping 500 nL of mixture solution on the plate, 500 nL of matrix solution was deposited onto it and waited for drying. For LC ESI MS detection, 50 μL of mixed samples were derivatized by benzoyl chloride utilizing a standard procedure reported before^68^. Chromatography was performed on an Agilent Technologies Acquity UPLC system. Mass spectrometric detection was carried out using an Agilent Technologies Xevo G2-XS QTOFMS mass spectrometer equipped with an ESI source.

### Preparation of clinical samples

A total of 481 subjects were consecutively recruited from 2014 to 2019 in Shanghai Chest Hospital, including 200 patients suffering early-stage LA and 200 healthy controls undergoing routine health care maintenance, 36 patients with squamous carcinoma (including squamous cell carcinoma and small cell carcinoma), and 45 patients with benign lung diseases (including pneumonia, hamartoma, pulmonary tuberculosis, granuloma, and others). All patients were diagnosed by a panel of pathologists together and the tumours staged according to the international standards for TNM staging of lung cancer. The pathologists were blind to any information about the acquisition from MS analysis. Patients were excluded from the study if they had evidence of autoimmune syndromes or drugs. The blood was drawn at initial diagnosis without surgery or anaesthesia. All blood samples were drawn by venepuncture and clotted at room temperature within 40 min^16^. Serum samples were obtained by centrifuging at 5100×*g* and 4 °C for 10 min. After centrifugation, the precipitate was discarded and the supernatant serum was stored at −80 °C immediately (within 15 min). The elapsed time was within 1 h between blood draw, centrifugation, and ultimate storage at −80 °C^69^.

To validate the classification of early-stage LA and healthy controls, we recruited an independent double-blind test cohort from Shanghai Chest Hospital, with serum samples from 58 subjects (23/35, early-stage LA/healthy controls). The situations for blood drawn were the same for all subjects.

All the investigation protocols in this study were approved by the institutional ethics committees of the Shanghai Chest Hospital and School of Biomedical Engineering, SJTU (KS1736). All subjects provided written informed consent to participate in the study and approved the use of their biological samples for analysis, according to the Helsinki Declaration.

### Machine learning and computer-aided diagnosis

Considering the large size of MS data, the sparse learning and regression model was employed for the diagnosis of subjects. Models generated can be simpler to interpret duet to the “sparse” models (involving only a subset of the features). Given a set of training subjects, we defined the matrix **X** = {⋯, **x**_*i*_,⋯}, where each row recorded the serum metabolic patterns (mass spectra) of the corresponding subject. The disease labels (i.e., ‘1’ for early-stage LA, ‘0’ for healthy control) of the training subjects were known already and were vectorized into the column vector $\overrightarrow {\mathbf{y}} = \left( { \cdots ,\overrightarrow {{\mathbf{y}}_{\mathbf{i}}} , \cdots } \right)\prime$ accordingly. The *l*_1_-norm (and the squared *l*_2_-norm) regularized logistic regression model could thus be acquired by solving the following:

1 $$\min _{\overrightarrow \beta ,c}\mathop {\sum }\limits_{i = 1}^m \ln \left( {1 + {\mathrm{{e}}}^{ - \overrightarrow {\mathbf{y}} _i\left( {x_i\overrightarrow {\beta} + c} \right)}} \right) + \frac{{\lambda _1}}{2}\left\| {\overrightarrow {\beta} } \right\|_{l_1} + \lambda _2\left\| {\overrightarrow {\beta} } \right\|_{l_2}$$

where *λ*_1_ was the *l*_1_-norm regularization parameter enforcing the sparsity constraint, and *λ*_2_ was the regularization parameter for the squared *l*_2_-norm. The model chose a limited number of *m*/*z* features by adjusting *l*_1_-norm to attenuate the coefficients of the less significant features to 0, and fit the disease labels of the training subjects according to the selected *m*/*z* features. A mathematical weight for each statistically informative feature was calculated depending on the importance of the mass spectral feature in differentiating early-stage LA versus healthy control. The regression model was applicable to infer the disease label of a new test subject and provided a prediction score for each pattern of a test sample. Specifically, we detected **x**_test_ and computed $\overrightarrow {\mathbf{y}} _{{\mathrm{test}}} = {\mathbf{x}}_{{\mathrm{test}}}^\prime \cdot \vec \beta + c$. The outcome was thresholded and converted to a diagnosis.

For a typical machine-learning-based diagnosis, five mass spectra obtained for each sample were used to build molecular databases. Pre-processing of the raw mass spectra data, including baseline correction, peak detection, extraction, alignment, normalization, and standardization, was carried out by MATLAB (R2016a, The MathWorks, Natick, MA) prior to pattern recognition analysis. The total number of metabolite signals for each mass spectrum was detected, and then, *m*/*z* features were selected based on the Otsu algorithm and utilized in the subsequent analysis.

To build the classifier model and evaluate the performance, a five-fold cross-validation approach was performed to estimate the performance of the predictor for both the inner-loop and outer cross-validation (20 rounds for each fold, thus 100 models for outer cross-validation in total). The performance of the classifiers was measured based on the receiver operation curve (ROC) by the area under curve (AUC), calculating the proportions of concordant pairs among all pairs of observations, with 1 indicating perfect prediction accuracy.

To validate the discriminant performance of the built classifier on an external double-bind test cohort for differentiating early-stage LA from healthy controls, 58 samples (23/35: LA/healthy controls) were enrolled. The disease labels of the double-bind test cohort were unknown and predicted by the classifier. Further comparing the predicted disease labels with the true disease status, we computed the sensitivity, specificity, and AUC. A step-by-step protocol describing the preparation of ferric particles, MS data acquisition, clinical sample preparation, and computer-aided diagnosis can be found at Nature Protocol Exchange^70^.

### Potential biomarker identification

To identify the metabolic panel that contributed the most to diagnosis, two major aspects were considered for the 100 tuned models. First, we ranked the *m*/*z* features according to the model selected frequency and chose the top *m*/*z* features with repeat occurrence over 90% in 100 models. In parallel, we selected *m*/*z* features with a *p*-value < 0.05 according to two-sided Student’s *t*-test. Verification of the metabolites that were both frequently occurring and displayed a significant difference between early-stage LA and healthy control was conducted manually by *m*/*z* feature selection using the human metabolome database (HMDB, http://www.hmdb.ca/) and subsequent validation by tandem MS and accurate mass measurement (for both Na^+^-adducted and K^+^-adducted signals). Pearson correlations were computed between the Na^+^-adducted and K^+^-adducted signals of metabolites. The differential metabolomic profiles reflecting their respective biochemical pathways were analysed by MetaboAnalyst (http://www.metaboanalyst.ca/).

### Statistical analysis

Multivariate statistics were performed using the SIMCA software package (version 14.0, Umetrics, Umeå, Sweden). Before analysis, all mass spectra were scaled to Pareto (par) by dividing variables using the square root of the standard deviation when centring was completed. All covariates were tested, including age and sex. Logistic regression model was fit to evaluate the association of metabolic biomarkers with the presence of early-stage LA. Odds ratios with 95% confidence interval (CI) were calculated for metabolic biomarkers (including histamine, uracil, cysteine, HPA, UA, IA, and FA (18:2)). Before the analysis, all metabolites were centred and standardized to have a mean of 0 and a standard deviation of 1. Age and sex were added as covariates to the basic logistic regression model to calculate the adjusted odds ratios. An unsupervised principal component analysis (PCA) model was constructed from a number of principal components (PCs, orthogonal transformation of *m*/*z* features into linearly uncorrelated variables). All the statistical models above were manually optimized. The transformation was defined that the first PC accounted for the largest variance (as much of the variability in the dataset as possible). From the results of PCA analysis, we can obtain a PCA score plot, by visualizing the first two PCs in a two-dimensional space. To quantify the reproducibility of clinical serum samples, the *p* value for the normal distribution test (Lilliefors (Kolmogorov–Smirnov) test) was acquired through the *lillietest* function in MATLAB, with the null hypothesis at the default 5% significance level.

Power analysis was performed by uploading 12 samples (6/6: LA patients/healthy controls) as the pilot metabolomic data into MetaboAnalyst at a FDR of 0.1. As the result, the predicted power for estimating the effect sample size was set as 0.8^40,41^. To investigate the spectra similarity within one group, we computed the similarity scores for each group (both early-stage LA and healthy controls). Typically, one experimental spectrum obtained from a serum sample for different cohorts was randomly selected and fixed as the reference spectrum. The other experimental spectra within the same cohort were compared with the reference spectrum, and spectral similarity scores were calculated. The similarity score between two mass spectra (*i* and *j*) was calculated by cosine correlation method following a reported algorithm^44^ defined as

2 $${\mathrm{{cos}}} = \frac{{\overrightarrow {{\mathbf{y}}_{\boldsymbol{i}}} \cdot \overrightarrow {{\mathbf{y}}_{\boldsymbol{j}}} }}{{\left| {\overrightarrow {{\mathbf{Y}}_{\boldsymbol{i}}} } \right| \cdot \left| {\overrightarrow {{\mathbf{Y}}_{\boldsymbol{j}}} } \right|}} = \frac{{\mathop {\sum }\nolimits_{k = 1}^l y_{ik}y_{jk}}}{{\sqrt {\mathop {\sum }\nolimits_{t = 1}^{n_i} Y_{it}^2} \cdot \sqrt {\mathop {\sum }\nolimits_{t = 1}^{n_j} Y_{jt}^2} }}$$

where *y* was the normalized intensity of a peak appearing in both spectrum *i* and spectrum *j* (an identical peak), *l* was the number of identical peaks in the two spectra, *Y* was the normalized intensity of a peak appearing in a spectrum and *n* was the number of peaks in a spectrum.

Other statistical analyses in this work were performed by using SPSS software (version 19.0, SPSS Inc., USA) to calculate the *p* value for statistical demonstration, including two-sided Student’s *t*-test and one-way ANOVA. All significance level was set as 5%. Specifically, the means comparison in one-way ANOVA was based on Bonferroni corrections.

### Reporting summary

Further information on research design is available in the Nature Research Reporting Summary linked to this article.

## Supplementary information

Supplementary Information

Reporting summary

## Data Availability

The verification of the metabolites in this study was achieved by comparing the *m*/*z* features with human metabolome database (HMDB, http://www.hmdb.ca/). The data that support the findings of this study are available from the corresponding author upon reasonable request. Source data are provided with this paper.

## Source data

Source Data

## Code availability

The custom computer codes utilized during the current study are available from the corresponding author upon reasonable request, due to the competing financial interests. Source data are provided with this paper.
