# Supplementary material for: Machine learning of serum metabolic patterns encodes early-stage lung adenocarcinoma
Source: Nat Commun. 2020 Jul 16;11:3556. doi: 10.1038/s41467-020-17347-6 (PMC7366718; doi:10.1038/s41467-020-17347-6)
Supplement: Supplementary file 1 — Supplementary Information [file 41467_2020_17347_MOESM1_ESM.pdf]

Supplementary Information

Machine learning of serum metabolic patterns encodes early-stage lung adenocarcinoma

Huang et al.

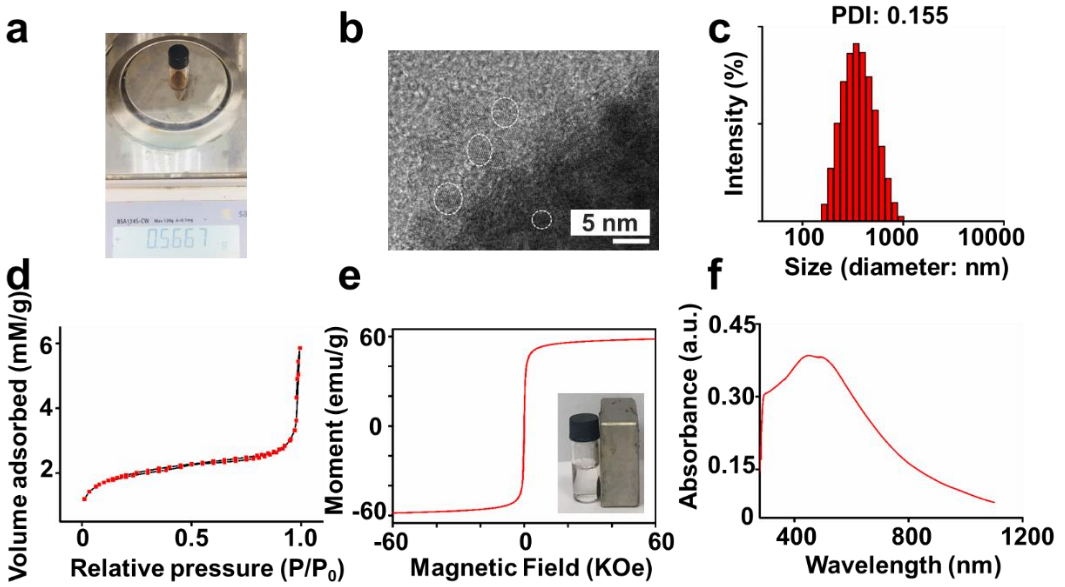

Supplementary Fig. 1. (a) Ferric particles from a single synthesis experiment, weight 0.567 g. (b) High-resolution transmission electron microscopy (HR-TEM) image of ferric particles ($n \geqslant 3$ randomly selected). Scale bar = 5 nm. The typical crystalline lattice is outlined by white circles. (c) Size distribution of ferric particles at 25°C in water by dynamic light scattering (DLS). (d) Typical nitrogen adsorption isotherm of ferric particles to identify crevices on the surface of particles (n = 55 data collection points). The sample was degassed before the experiment. (e) Magnetic hysteresis loop of ferric particles at 300 K, to show superparamagnetic properties. Inset image shows ferric particles separated by a permanent magnet. (f) Absorption spectrum of ferric particles. Source data are provided as a Source Data file.

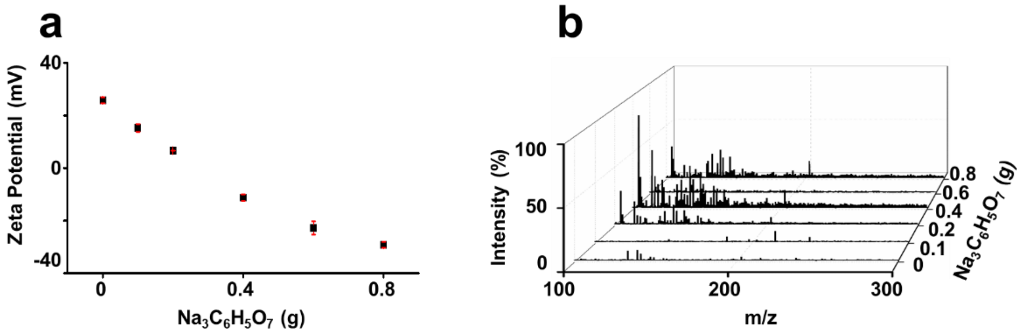

Supplementary Fig. 2. Optimization and application of ferric particles. (a) The relationship between the concentration of trisodium acetate and the surface charge of ferric particles. By varying the concentration of trisodium citrate (0-0.8 g) during the material synthesis the surface charge could be adjusted from zeta potential +27.3 eV to -30.3 eV, and the results showed good linearity with R^2=0.958. Three independent experiments were performed for each concentration to calculate the standard deviation (s.d.) as error bars. Data were shown as the mean ± s.d. (n = 3) (b) Serum metabolic pattern (mass spectra) from a healthy volunteer using ferric particles synthesized with different amounts of trisodium acetate. Source data are provided as a Source Data file.

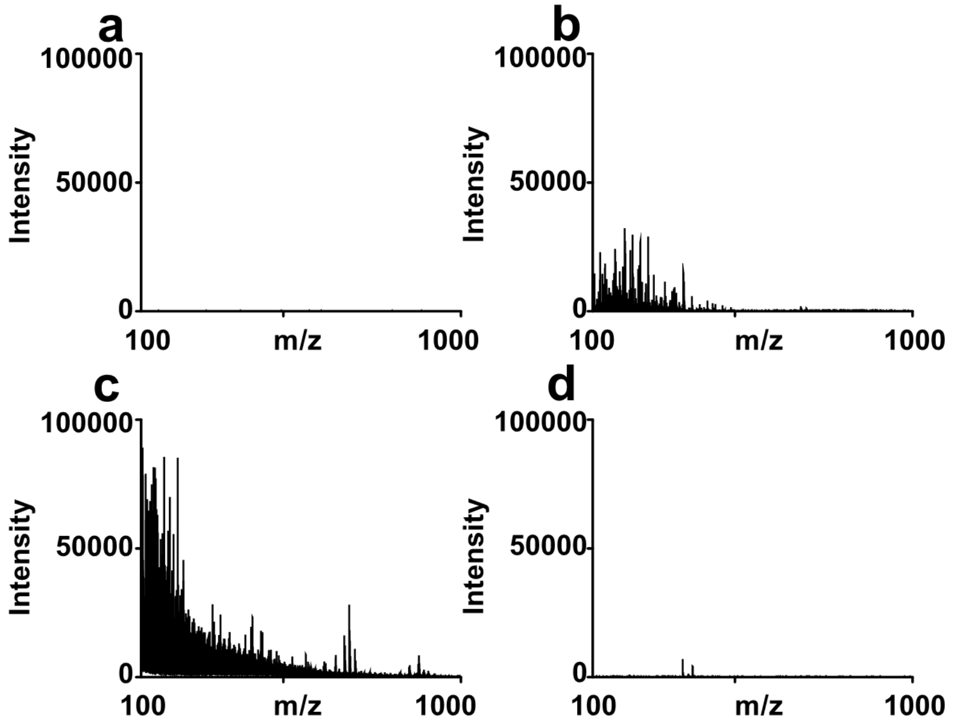

Supplementary Fig. 3. Serum detection using other matrices. Laser desorption/ionization (LDI) mass spectra of 500 nL of native serum using (a) no matrix, (b) CHCA, (c) carbon nanoparticles, and (d) silica nanoparticles in the positive ion mode. Source data are provided as a Source Data file.

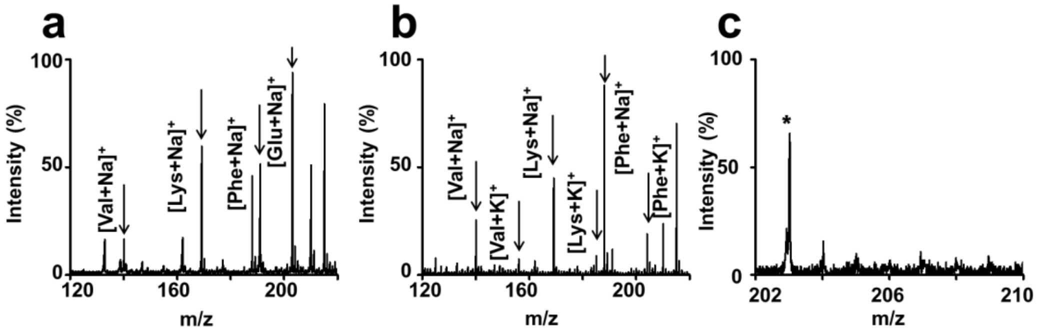

Supplementary Fig. 4. Salt tolerence, protein endurance, and serum detection of ferric particles. Mass spectra from ferric particle-assisted LDI mass spectrometry (MS) using samples of (a) 2 nmol valine (Val), lysine (Lys), phenylalanine (Phe), and glucose (Glu) in 0.5 M NaCl and (b) 2 nmol valine, lysine, and phenylalanine in 5 mg mL^{-1} bovine serum albumin solution and 0.5 M NaCl with sodium or potassium adductions, demonstrating the salt tolerance and protein endurance of ferric particles in LDI MS. (c) Ferric particle-assisted LDI MS spectrum of 500 pL of unpretreated serum. For clarity, only glucose (asterisk) is shown. Source data are provided as a Source Data file.

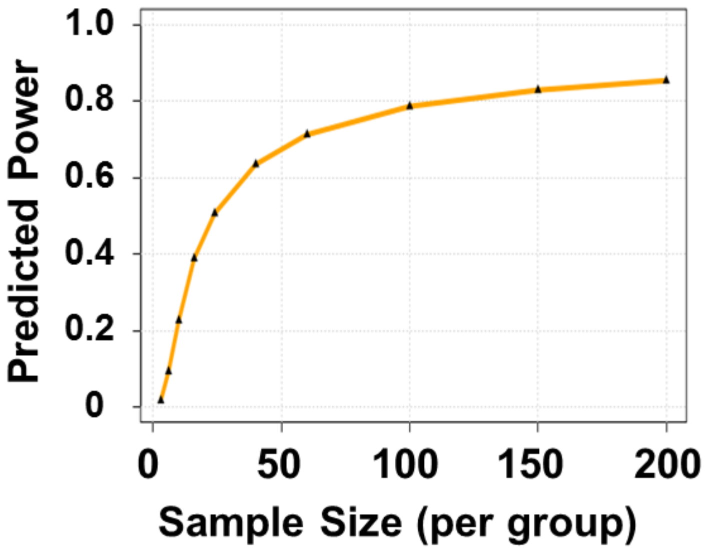

Supplementary Fig. 5. Power analysis of pilot data. The metabolic patterns extracted from 12 samples (6/6, lung adenocarcinoma (LA)/healthy control) were analysed to compute the minimum number of samples required with meaningful machine learning. Minimum sample size of 100 for each group will achieve the predicted power ~0.8, which can be at sufficient confidence level to conclude the statistical meaningful results. Source data are provided as a Source Data file.

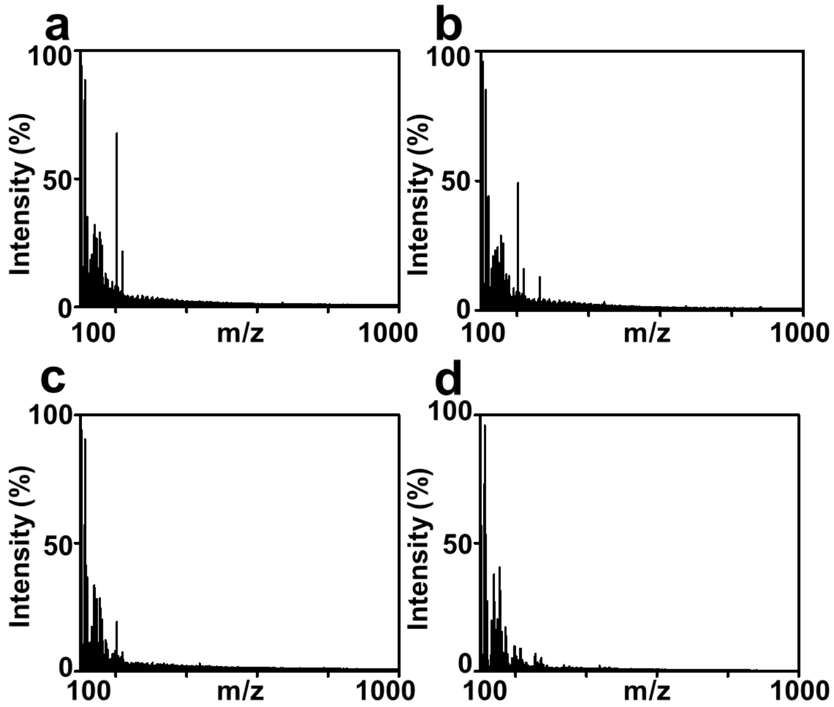

Supplementary Fig. 6. Typical serum metabolic patterns (mass spectra) with m/z ranging from 100 to 1000 by optimized ferric particles assisted LDI MS of (a) a patient with early-stage LA, (b) a healthy volunteer, (c) a patient with benign lung disease, and (d) a patient with squamous cell lung carcinoma. Source data are provided as a Source Data file.

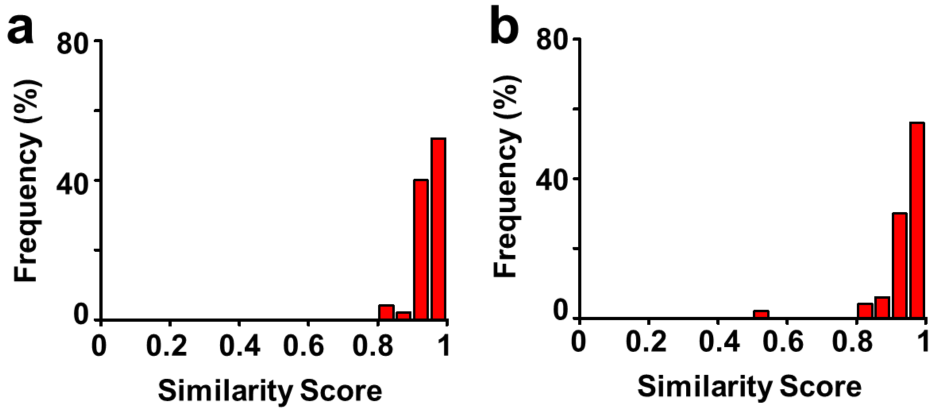

Supplementary Fig. 7. Frequency distribution of similarity scores. Frequency distribution of similarity scores were calculated for (a) early-stage LA and (b) healthy controls based on cosine correlation method. Source data are provided as a Source Data file.

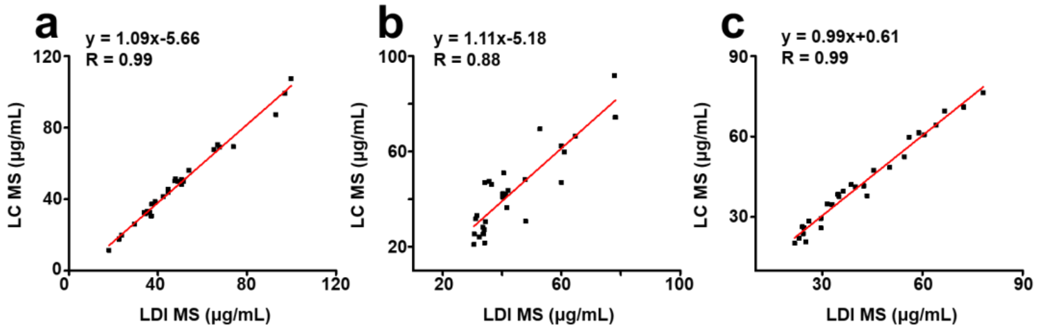

Supplementary Fig. 8. Linear correlation between liquid chromatography electrospray ionization (LC ESI MS) and LDI MS. Quantification results for samples consisted of different content of (a) glucose, (b) histamine, and (c) mannitol, affording R of 0.88-0.99 (n = 28 independent mixed samples).

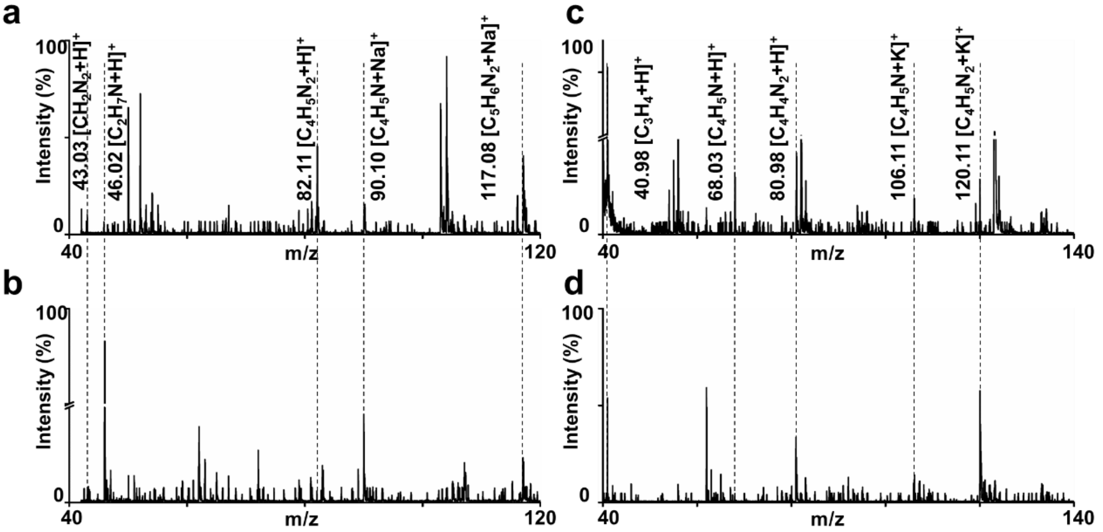

Supplementary Fig. 9. Tandem MS of Na^+- and K^+-adducted histamine. MS/MS of histamine at m/z 134.09 for $[M+Na]^+$ in (a) standard histamine sample in aqueous solution and (b) serum sample from an early-stage LA patient. MS/MS of histamine at m/z 150.09 for $[M+K]^+$ in (c) standard histamine sample in aqueous solution and (d) serum sample from an early-stage LA patient. Signals of fragment ions, as marked, were matched with the human metabolome database (HMDB, http://www.hmdb.ca/). Source data are provided as a Source Data file.

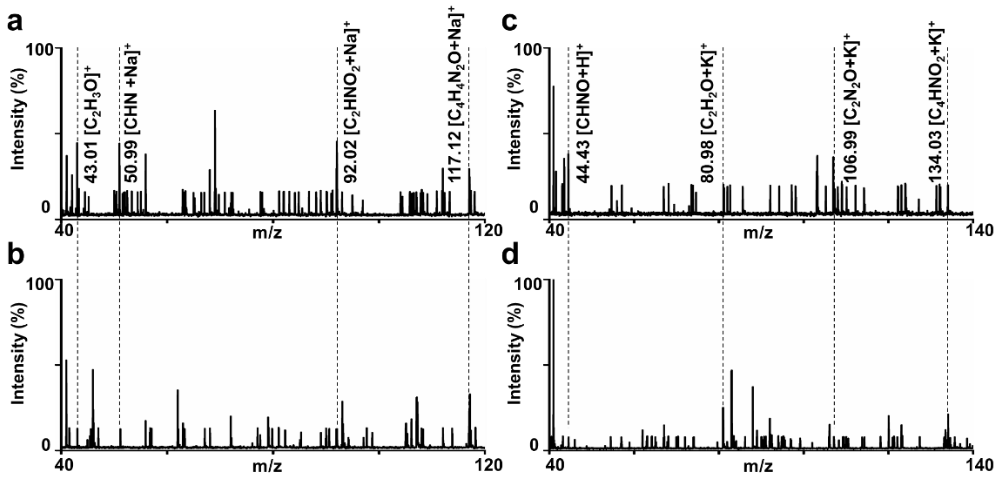

Supplementary Fig. 10. Tandem MS of Na^+- and K^+-adducted uracil. MS/MS of uracil at m/z 135.06 for $[M+Na]^+$ in (a) standard uracil sample in aqueous solution and (b) serum sample from an early-stage LA patient. MS/MS of uracil at m/z 151.06 for $[M+K]^+$ in (c) standard uracil sample in aqueous solution and (d) serum sample from an early-stage LA patient. Signals of fragment ions as marked were matched with the human metabolome database (HMDB, http://www.hmdb.ca/). Source data are provided as a Source Data file.

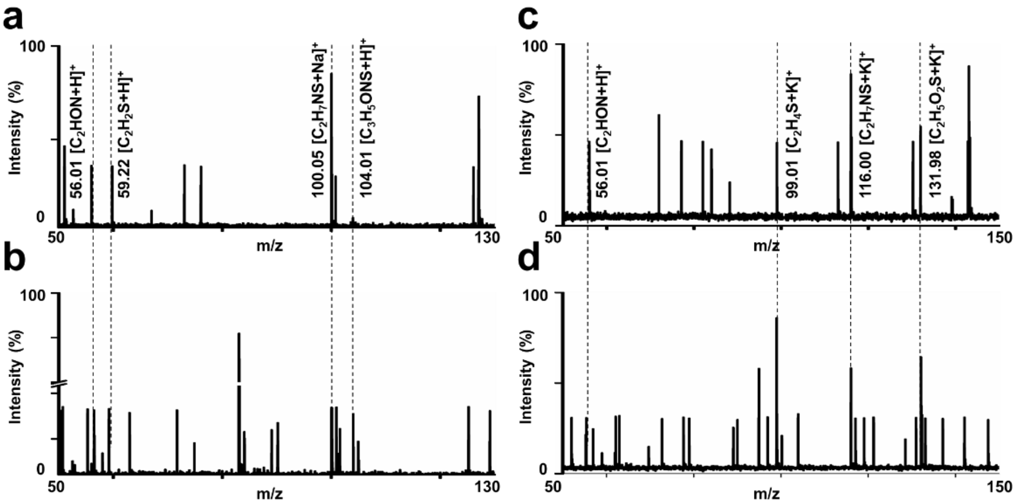

Supplementary Fig. 11. Tandem MS of Na^+- and K^+-adducted cysteine. MS/MS of cysteine at m/z 143.93 for $[M+Na]^+$ in (a) standard cysteine sample in aqueous solution and (b) serum sample from an early-stage LA patient. MS/MS of cysteine at m/z 160.03 for $[M+K]^+$ in (c) standard cysteine sample in aqueous solution and (d) serum sample from an early-stage LA patient. Signals of fragment ions as marked were matched with the human metabolome database (HMDB, http://www.hmdb.ca/). Source data are provided as a Source Data file.

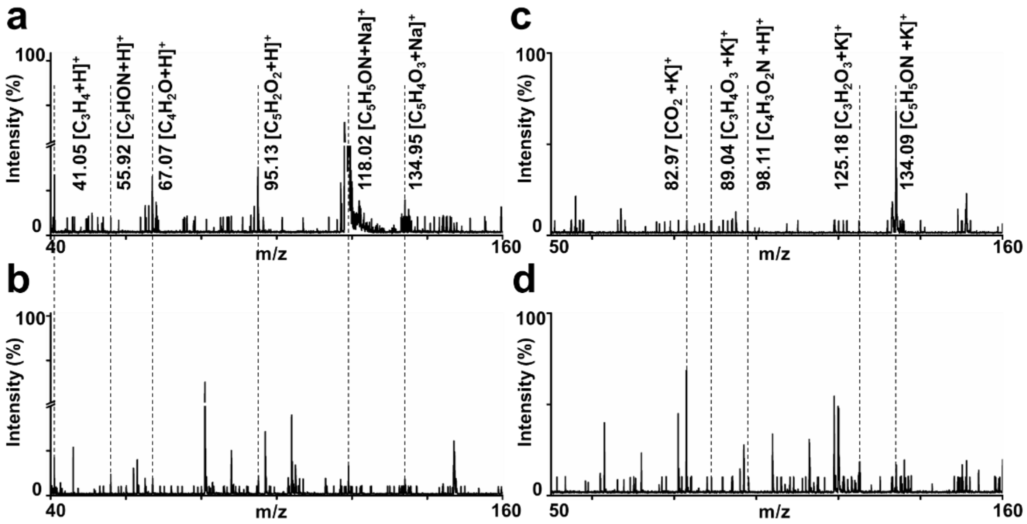

Supplementary Fig. 12. Tandem MS of Na^+- and K^+-adducted 3-hydroxypicolinic acid (HPA). MS/MS of HPA at m/z 162.11 for $[M+Na]^+$ in (a) standard HPA sample in aqueous solution and (b) serum sample from an early-stage LA patient. MS/MS of HPA at m/z 178.11 for $[M+K]^+$ in (c) standard HPA sample in aqueous solution and (d) serum sample from an early-stage LA patient. Signals of fragment ions as marked were matched with the human metabolome database (HMDB, http://www.hmdb.ca/). Source data are provided as a Source Data file.

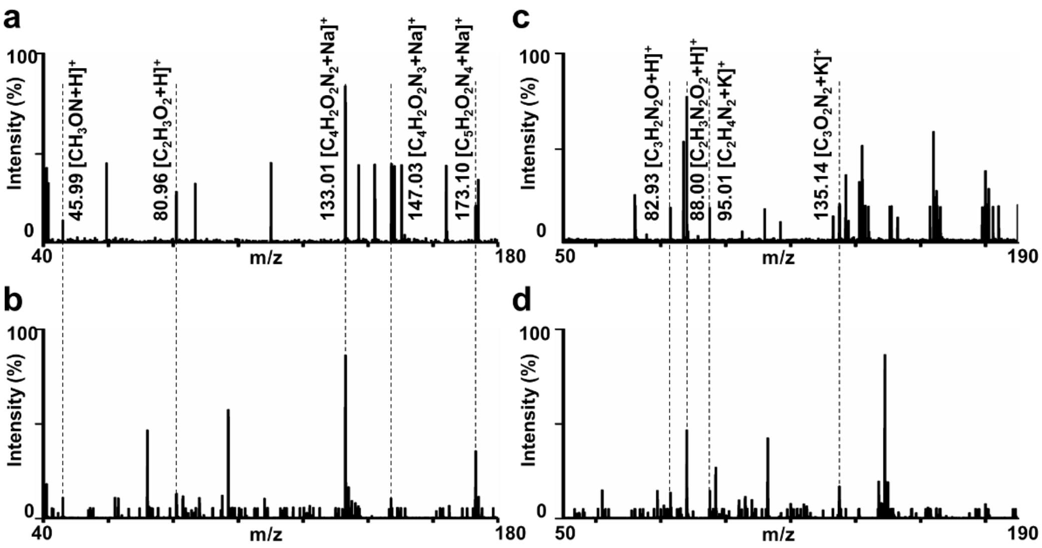

Supplementary Fig. 13. Tandem MS of Na^+- and K^+-adducted uric acid. MS/MS of uric acid at m/z 191.06 for $[M+Na]^+$ in (a) standard uric acid sample in aqueous solution and (b) serum sample from an early-stage LA patient. MS/MS of uric acid at m/z 207.06 for $[M+K]^+$ in (c) standard uric acid sample in aqueous solution and (d) serum sample from an early-stage LA patient. Signals of fragment ions as marked were matched with the human metabolome database (HMDB, http://www.hmdb.ca/). Source data are provided as a Source Data file.

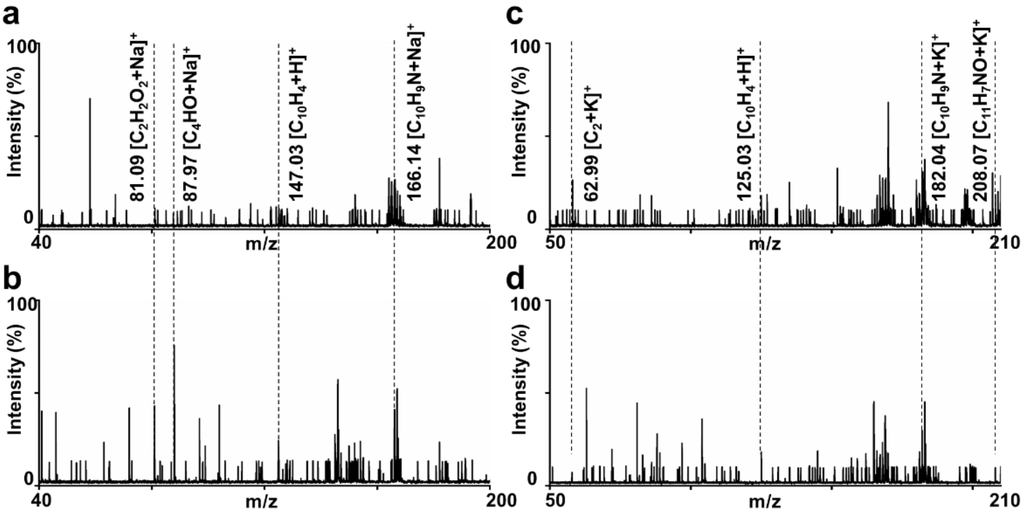

Supplementary Fig. 14. Tandem MS of Na^+- and K^+-adducted indoleacrylic acid (IA). MS/MS of IA at m/z 210.12 for $[M+Na]^+$ in (a) standard IA sample in aqueous solution and (b) serum sample from an early-stage LA patient. MS/MS of IA at m/z 226.12 for $[M+K]^+$ in (c) standard IA sample in aqueous solution and (d) serum sample from an early-stage LA patient. Signals of fragment ions as marked were matched with the human metabolome database (HMDB, http://www.hmdb.ca/). Source data are provided as a Source Data file.

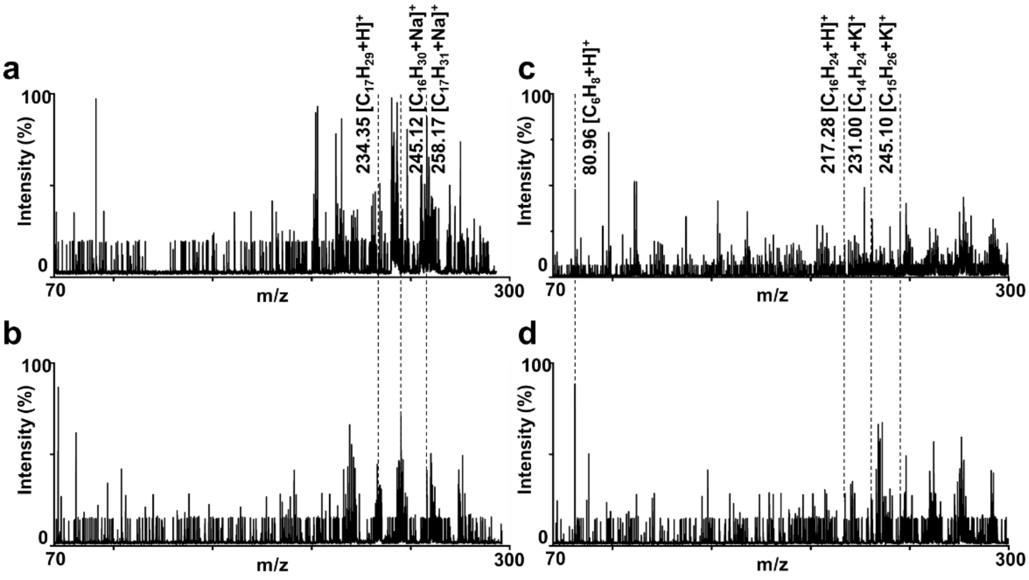

Supplementary Fig. 15. Tandem MS of Na^+- and K^+-adducted fatty acid (FA) (18:2). MS/MS of FA at m/z 303.34 for $[M+Na]^+$ in (a) standard FA sample in aqueous solution and (b) serum sample from an early-stage LA patient. MS/MS of FA at m/z 319.34 for $[M+K]^+$ in (c) standard FA sample in aqueous solution and (d) serum sample from an early-stage LA patient. Signals of fragment ions as marked were matched with the human metabolome database (HMDB, http://www.hmdb.ca/). Source data are provided as a Source Data file.

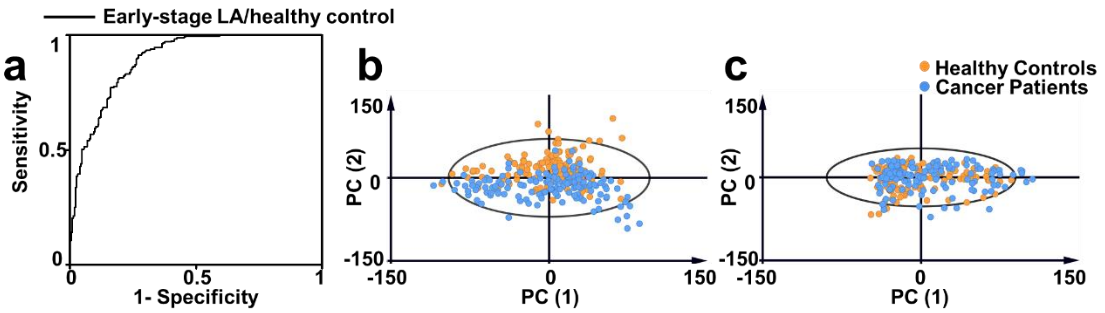

Supplementary Fig. 16. Receiver operating characteristic (ROC) curve (a) using the metabolic biomarker panel for differentiating early-stage LA from healthy control (area under the curve (AUC) = 0.894). Principal component analysis (PCA) score plots of (b) seven selected metabolites and (c) all 161 m/z features with the first two PCs explaining 73.71% and 67.28% of the total variance, respectively. Source data are provided as a Source Data file.

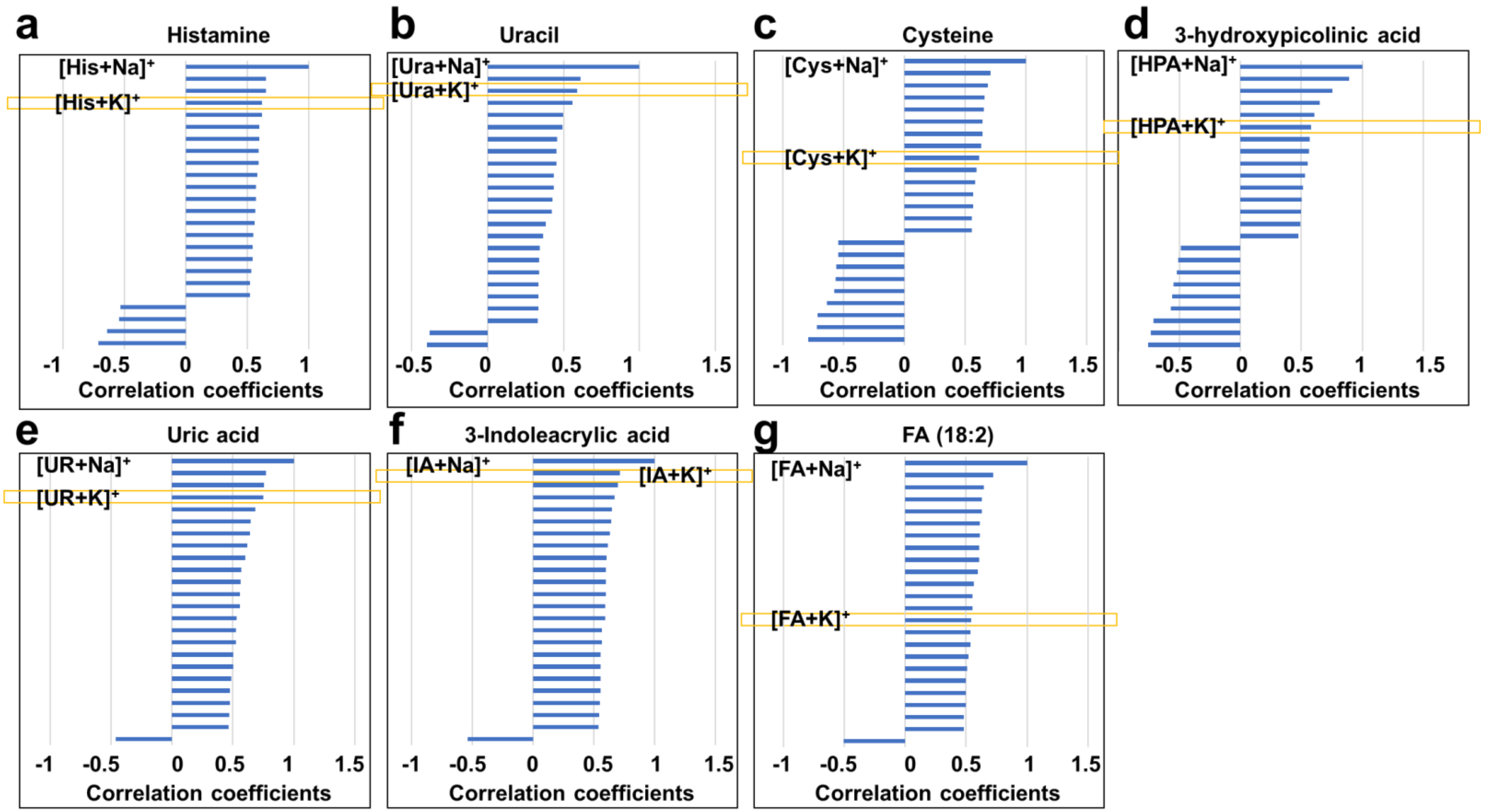

Supplementary Fig. 17. Top 25 signals correlated with Na^+-adducted (a) histamine, (b) uracil, (c) cysteine, (d) 3-hydroxypicolinic acid, (e) uric acid, (f) indoleacrylic acid, and (g) fatty acid (FA) (18:2) for early-stage LA and healthy volunteers (the first bar in each figure stood for the peak intensity of Na^+-adducted signals with correlation coefficients as 1) according to Pearson correlation. The yellow boxes indicate K^+-adducted signals. Source data are provided as a Source Data file.

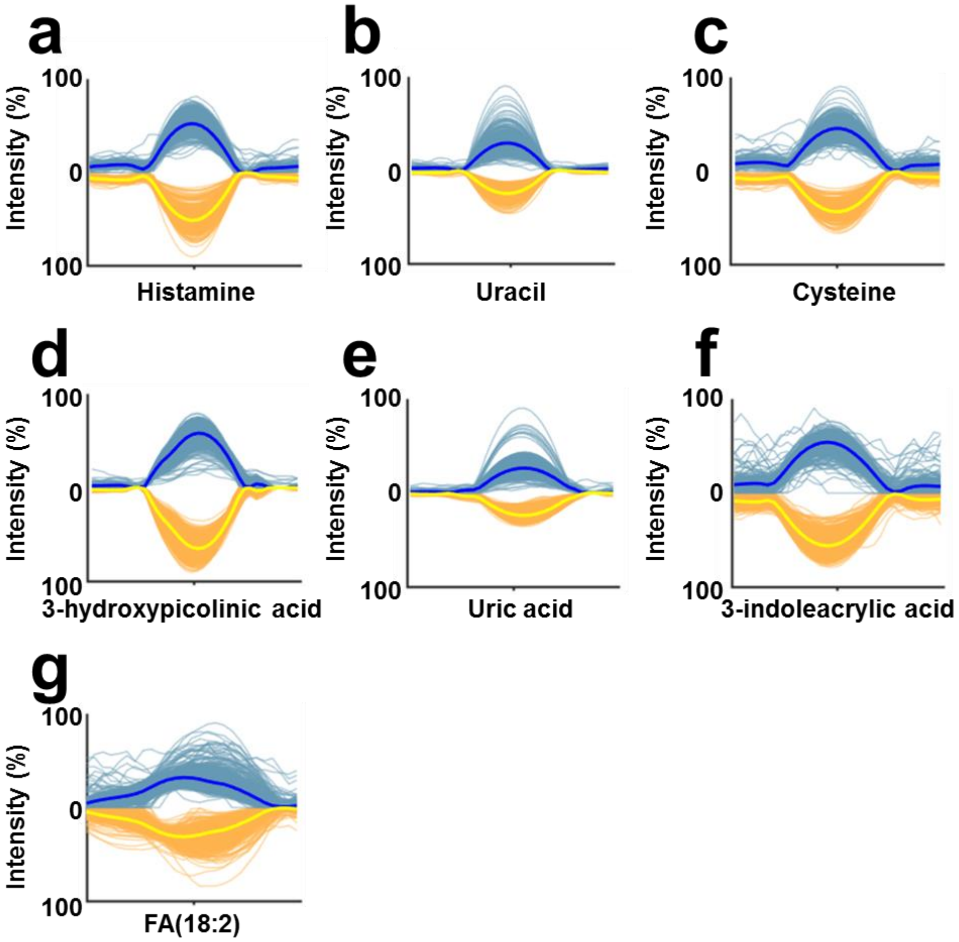

Supplementary Fig. 18. Localized mass spectra for each biomarker in the serum metabolic patterns, including (a) histamine, (b) uracil, (c) cysteine, (d) 3-hydroxypicolinic acid, (e) uric acid, (f) indoleacrylic acid, and (g) fatty acid (FA) (18:2) for early-stage LA (blue) and healthy controls (yellow). The bold blue and yellow lines refer to the overall averaged mass spectra from early-stage LA and healthy controls, respectively. Source data are provided as a Source Data file.

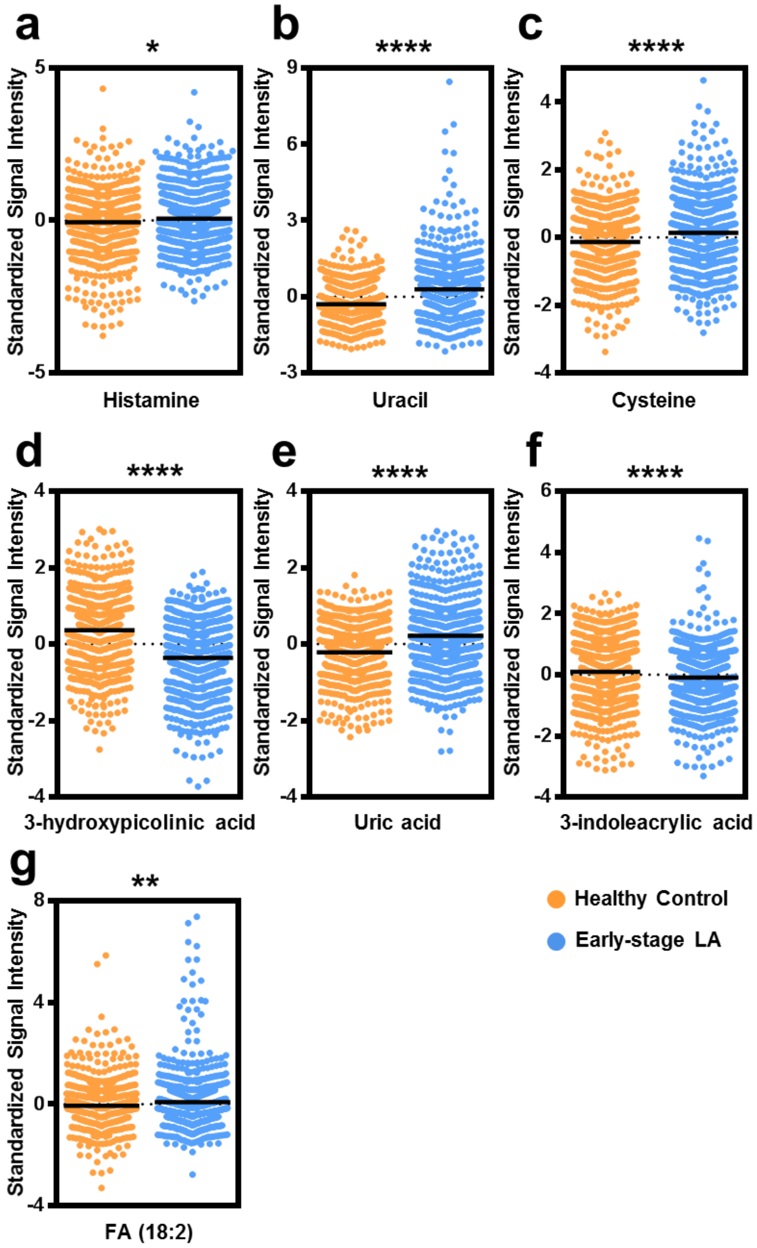

Supplementary Fig. 19. Scatter plots for z score standardized signal intensities of seven metabolites for early-stage LA patients (blue) and healthy controls (yellow), including (a) histamine (p = 0.03), (b) uracil (p = 5.06E-30), (c) cysteine (p = 1.91E-07), (d) 3-hydroxypicolinic acid (p = 4.16E-48), (e) uric acid (p = 2.25E-17), (f) indoleacrylic acid (p = 0.00018), and (g) fatty acid (FA) (18:2) (p = 0.00528), calculated by converting all m/z features to a scale with an average of 0 and standard deviation of 1 (* $p < 0.05$; ** $p < 0.01$; **** $p < 0.0001$, n = 300 biologically independent samples, two-sided Student's t-test with no adjustment made for multiple comparisons). Source data are provided as a Source Data file.

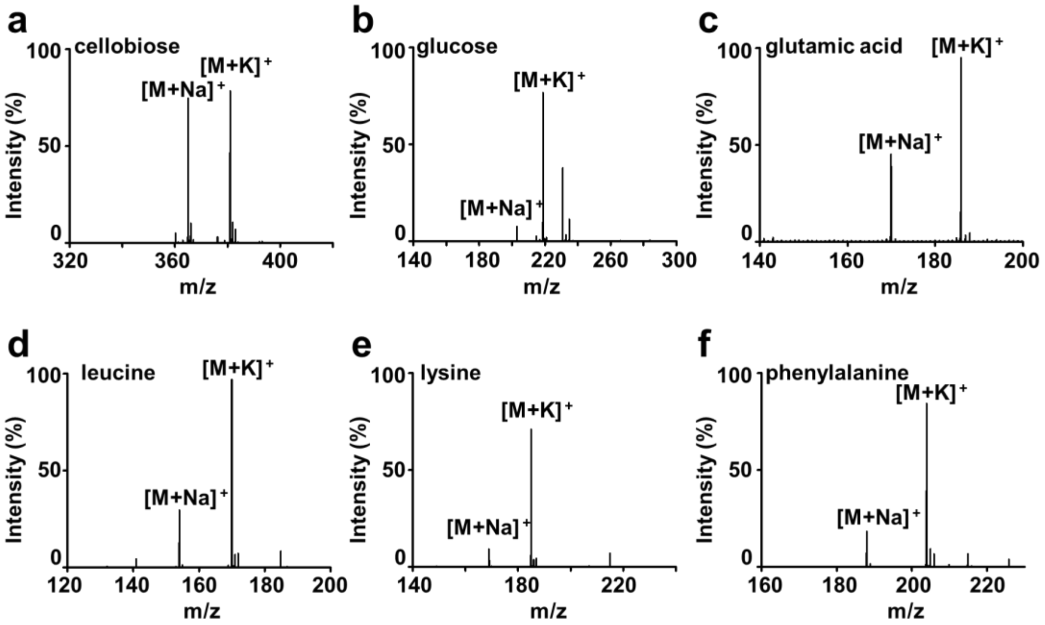

Supplementary Fig. 20. Typical mass spectra of 10 ng μL^{-1} (a) cellobiose, (b) glucose, (c) glutamic acid, (d) leucine, (e) lysine, and (f) phenylalanine in the positive ion mode, showing sodium and potassium adducted molecular peaks using ferric particle as matrix. Source data are provided as a Source Data file.

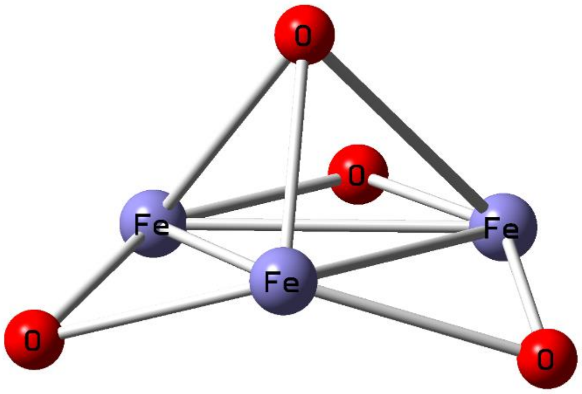

Supplementary Fig. 21. Density functional theory (DFT) calculation model of ferric particles on the exposed surface [1,1,1].

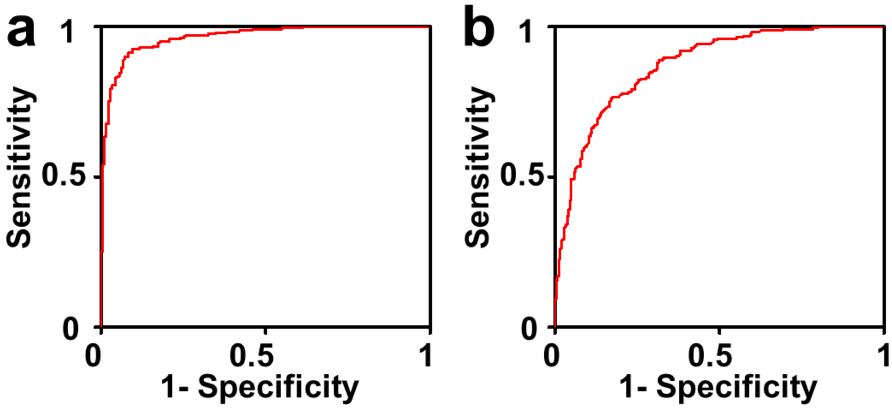

Supplementary Fig. 22. ROC curves for differentiating early-stage LA from (a) other lung cancers (AUC = 0.963) and (b) benign diseases (AUC = 0.863) using the metabolic biomarker panels identified in the corresponding disease classifiers. The metabolic biomarker panels were identified based on both the repeat occurrence (frequency > 90%) and statistical significance ($p < 0.05$, the exact p value provided in Supplementary Table 7&8). Source data are provided as a Source Data file.

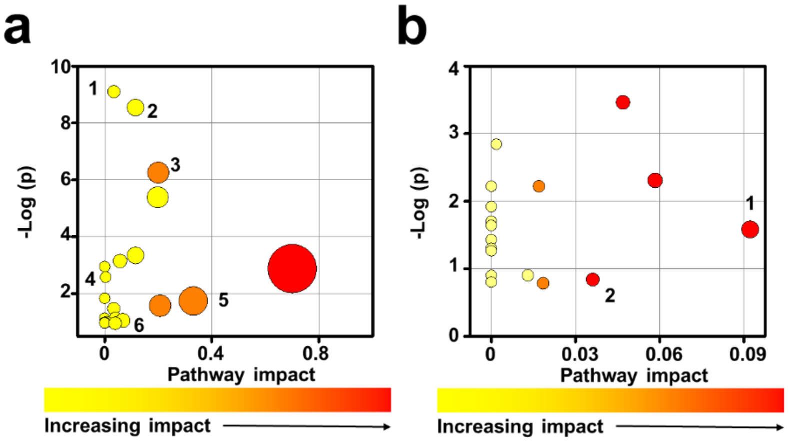

Supplementary Fig. 23. Potential pathways differentially regulated in (a) other lung cancer and (b) benign diseases. The colour and size of each circle were correlated to the *p* value and pathway impact value. Pathways with impact values > 0 were considered to be differentially altered. The shared metabolic pathways in early-stage LA and (a) is (1) beta-alanine metabolism, (2) pyrimidine metabolism, (3) pantothenate and CoA biosynthesis, (4) glycine, serine, and threonine metabolism, (5) taurine and hypotaurine metabolism, and (6) histidine metabolism. The shared metabolic pathways in early-stage LA and (b) is (1) histidine metabolism and (2) pyrimidine metabolism.

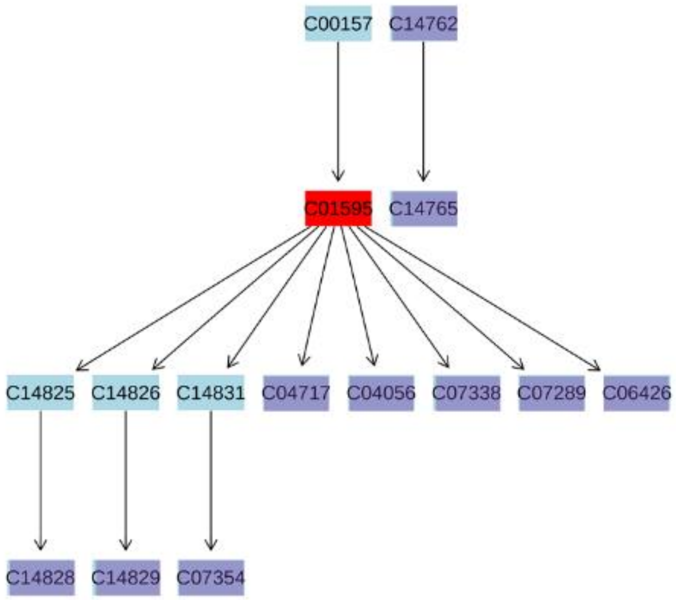

Supplementary Fig. 24. Schematic illustration of FA metabolism. The compound colours within the pathway - blue represented metabolites that were not in the data and used as background in topology analysis with total importance of 0.34; purple represented metabolites that were not in the data and used as background in topology analysis with total importance of 0; and red represented the metabolite (FA) that was in the data and used in topology analysis with total importance of 0.66.

Supplementary Table 1. Baseline characteristics of patients and healthy volunteers.

Disease	Subtypes	Number		Sex		Age (median(range))	F	*p* value	Smoking history		Stage[a]					
		Subtypes	Total	Female	Male				Yes	No	I	II	III	IV	Extensive	Limited
Healthy control	NA	200	200	100	100	53 (32-71)	0.088	0.767	NA	NA	NA	NA	NA	NA	NA	NA
Lung adenocarcinoma	NA	200	200	100	100	54 (32-71)			13	187	200	0	0	0	NA	NA
Squamous carcinoma	Squamous cell carcinoma	18	36	3	15	54 (44-72)			22	14	7	2	7	2	NA	NA
	Small cell carcinoma	18		2	16						NA	NA	NA	NA	6	12
Benign disease	Pneumonia	15	45	10	5	52 (33-71)			NA	NA	NA	NA	NA	NA	NA	NA
	Hamartoma	1		1	0											
	Pulmonary tuberculosis	12		6	6											
	Granuloma	14		7	7											
	Others	3		1	2											

[a] The disease stage is based on the international standard in tumor, node, and metastasis (TNM) staging of lung cancer.

Supplementary Table 2. Consistency between standard LC ESI MS method and ferric particles assisted LDI MS approach.

Sample number	Glucose (μg mL^{-1})	Glucose detected by LDI MS (μg mL^{-1})	Glucose detected by LC ESI MS (μg mL^{-1})	Histamine (μg mL^{-1})	Histamine detected by LDI MS (μg mL^{-1})	Histamine detected by LC ESI MS (μg mL^{-1})	Mannitol (μg mL^{-1})	Mannitol detected by LDI MS (μg mL^{-1})	Mannitol detected by LC ESI MS (μg mL^{-1})
1	50.00	51.55	49.81	41.67	40.20	42.23	41.67	39.94	41.16
2	17.39	22.51	17.59	86.96	77.84	91.83	26.09	29.59	25.88
3	57.14	53.93	55.9	28.57	33.90	27.21	38.10	34.68	38.46
4	52.17	50.64	51.1	43.48	41.15	41.92	34.78	32.86	34.65
5	45.28	44.65	45.54	22.64	30.58	25.31	71.70	72.38	70.99
6	26.09	29.52	26.13	43.48	47.69	48.08	60.87	60.53	60.67
7	52.17	47.95	51.39	52.17	40.48	50.93	26.09	24.58	25.97
8	103.45	99.86	107.39	34.48	34.37	30.48	20.69	23.06	21.98
9	38.10	37.30	37.14	47.62	36.41	46.21	38.10	35.02	37.65
10	42.11	42.46	41.46	31.58	31.09	31.69	42.11	42.42	41.56
11	10.53	18.03	11.41	42.11	35.53	47.43	63.16	58.91	61.39
12	69.57	65.15	67.68	26.09	33.65	25.31	34.78	31.50	34.81
13	30.77	23.71	19.69	30.00	31.41	33.05	70.00	66.67	69.55
14	69.57	67.64	68.96	34.78	47.89	30.72	26.09	24.04	26.31
15	96.55	97.03	99.05	20.69	30.47	20.9	27.59	25.98	28.50
16	85.71	93.08	87.24	21.43	34.06	21.63	50.00	54.65	52.50
17	32.00	34.42	31.97	56.00	60.91	59.84	48.00	50.20	48.42
18	30.77	37.02	30.46	69.23	78.17	74.29	38.46	43.47	37.85
19	50.00	47.68	50.11	25.00	32.29	24.11	58.33	55.97	59.80
20	32.00	33.76	32.57	40.00	41.96	43.69	64.00	64.13	64.35
21	32.00	36.43	32.36	56.00	59.92	62.42	48.00	45.42	47.37
22	50.00	48.94	49.75	41.67	40.21	40.88	41.67	38.64	42.05
23	68.97	73.91	69.31	55.17	59.99	47.03	20.69	21.74	20.17
24	33.33	35.31	33.12	25.00	33.45	28.08	75.00	78.22	76.28
25	40.00	38.77	38.47	40.00	41.63	36.34	40.00	36.33	39.52
26	48.00	50.62	48.1	64.00	64.63	66.37	24.00	24.28	23.74
27	44.44	44.71	43.86	66.67	52.74	69.39	29.63	29.57	29.43
28	68.97	66.83	70.32	55.17	34.20	46.93	20.69	25.03	20.76

Supplementary Table 3. Detection limit of standard metabolites by ferric particle-assisted LDI MS.

Analytes	Detection limit (pmol)
Glucose	28
Sucrose	1.5
Cellobiose	1.5
Mannitol	2.7
Leucine	38
Lysine	34
Methionine	34
Glutamic acid	34
Arginine	29

Supplementary Table 4. Baseline characteristics of patients and healthy volunteers for double-blind test.

Characteristics	Number	Sex		Age (median(range))	Smoking history		Stage[a]
		Female	Male		Yes	No	
Healthy control	35	22	13	45 (35-62)	NA	NA	NA
Lung adenocarcinoma	23	16	7	51 (29-72)	0	23	Ⅰ

[a] The disease stage is based on the international standard in TNM staging of lung cancer.

Supplementary Table 5. Seven metabolites selected as biomarkers to distinguish early-stage LA patients from healthy controls.

Metabolite	Molecular weight (Da)	Frequency (%)[a]	Coefficient[b]	*p* value[c]	AUC[d]
Histamine	111.14	100	0.40	0.03	0.51
Uracil	112.08	100	1.24	5.06E-30	0.67
Cysteine	121.15	100	0.22	1.91E-07	0.57
3-Hydroxypicolinic acid	139.11	100	0.37	4.16E-48	0.71
Uric acid	168.11	100	0.60	2.25E-17	0.61
Indoleacrylic acid	187.19	100	0.98	0.00018	0.56
Fatty acid (18:2)	280.45	100	0.16	0.00528	0.52

[a] Frequency referring to selection probability by optimized classifier for diagnosis in 100 models.
[b] Coefficient referring to sparsity constrained $\vec{\boldsymbol{\beta}}$.
[c] The *p* value was acquired for early-stage LA and healthy control through two-sided Student's t-test.
[d] AUC was acquired by univariate ROC curve analysis for the individual biomarker.

Supplementary Table 6. Odds ratios of metabolic biomarkers adjusted for age and sex.

Metabolite	Odds ratios (95% confidence interval (CI)) in basic model	Odds ratios (95% CI) in adjusted models with covariates		
		Age	Sex	Age and sex
Histamine	0.46 (0.38-0.56)	0.44 (0.36-0.54)	0.46 (0.37-0.56)	0.44 (0.35-0.54)
Uracil	2.81 (2.28-3.46)	3.10 (2.49-3.86)	2.89 (2.34-3.58)	3.10 (2.49-3.86)
Cysteine	1.84 (1.50-2.25)	1.90 (1.55-2.33)	1.85 (1.51-2.27)	1.90 (1.55-2.33)
3-Hydroxypicolinic acid	0.30 (0.25-0.35)	0.29 (0.25-0.35)	0.29 (0.25-0.35)	0.29 (0.25-0.35)
Uric acid	3.05 (2.33-3.99)	3.09 (2.36-4.04)	3.02 (2.31-3.95)	3.10 (2.36-4.06)
Indoleacrylic acid	0.45 (0.36-0.55)	0.43 (0.35-0.52)	0.44 (0.36-0.54)	0.43 (0.35-0.52)
Fatty acid (18:2)	1.27 (1.09-1.49)	1.28 (1.10-1.50)	1.30 (1.11-1.52)	1.28 (1.09-1.5)

Supplementary Table 7. Metabolites selected as biomarkers to distinguish early-stage LA from other lung cancers.

Metabolite	Molecular weight (Da)	Frequency (%)[a]	Coefficient[b]	*p* value[c]	AUC[d]
Pyridine	79.10	99	0.23	2.07E-36	0.87
2-Pyrrolidinone	85.10	96	0.04	6.61E-40	0.90
But-2-enoic acid	86.09	100	0.22	2.21E-44	0.91
2-Aminoacrylic acid	87.08	100	0.55	4.59E-49	0.92
Pyruvic acid	88.06	100	0.53	7.70E-42	0.89
D-Lactic acid	90.08	100	0.60	5.99E-33	0.81
Glycerol	92.09	99	1.65	1.70E-44	0.86
Purine	120.11	99	0.35	2.46E-31	0.84
D-Threitol	122.12	100	0.50	5.53E-46	0.88
D-Glutamine	146.14	99	0.19	5.79E-35	0.83
Uric acid	168.11	99	0.16	1.87E-10	0.65
Gallic acid	170.12	100	0.01	0.032	0.52
3-Phosphoglyceric acid	186.06	99	0.56	1.76E-20	0.75
Indoleacrylic acid	187.19	100	0.95	1.58E-06	0.62
Isovaleric acid	102.13	100	0.89	4.52E-50	0.93
Choline	104.17	97	0.16	5.73E-43	0.89
Malonic acid	104.06	100	0.67	2.46E-39	0.89
L-Glyceric acid	106.08	98	0.19	3.77E-44	0.89
Thymine	126.11	99	0.47	5.96E-55	0.92
Threonic acid	136.10	99	0.04	1.54E-24	0.79
Salicylic acid	138.12	100	0.72	9.58E-40	0.86

4-O-Methylgallic acid	184.15	99	0.22	4.27E-42	0.85

[a] Frequency referring to selection probability by optimized classifier for diagnosis in 100 models.
[b] Coefficient referring to sparsity constrained $\vec{\boldsymbol{\beta}}$.
[c] The *p* value was acquired for early-stage LA and other lung cancers through two-sided Student's t-test.
[d] AUC was acquired by univariate ROC curve analysis for the individual biomarker.

Supplementary Table 8. Metabolites selected as biomarkers to distinguish early-stage LA from benign diseases.

Metabolite	Molecular weight (Da)	Frequency (%)[a]	Coefficient[b]	*p* value[c]	AUC[d]
But-2-enoic acid	86.09	94	0.00	8.69E-10	0.71
2-Hydroxypyridine	95.10	97	0.05	4.78E-09	0.70
2,5-Dimethylfuran	96.13	100	0.68	2.30E-10	0.66
Acetoacetic acid	102.09	100	0.28	5.20E-09	0.70
Citraconic acid	130.10	99	0.21	6.20E-21	0.75
3-Hydroxypicolinic acid	139.11	100	0.57	3.91E-07	0.63
L-Theanine	174.19	100	0.10	6.74E-12	0.69
Galactosylceramide (d18:1/12:0)	643.94	99	0.49	0.052469	0.53
PS(14:1(9Z)/20:4(5Z,8Z,11Z,14Z))	753.94	100	0.48	0.026578	0.54

[a] Frequency referring to selection probability by optimized classifier for diagnosis in 100 models.
[b] Coefficient referring to sparsity constrained $\vec{\boldsymbol{\beta}}$.
[c] The *p* value was acquired for early-stage LA and benign diseases through two-sided Student's t-test.
[d] AUC was acquired by univariate ROC curve analysis for the individual biomarker.

Supplementary Table 9. Pathways regulated in early-stage LA and healthy controls.

Pathway Hit[a]	*p* value[b]	-log (p)	Impact[c]
Pantothenate and CoA biosynthesis	0.001768	6.3377	0
Fatty acid metabolism	0.036851	3.3009	0.65625
Sulphur metabolism	0.044084	3.1217	0.03307
Taurine and hypotaurine metabolism	0.04888	3.0184	0
Thiamine metabolism	0.058413	2.8402	0
beta-Alanine metabolism	0.067866	2.6902	0
Glutathione metabolism	0.091154	2.3952	0
Histidine metabolism	0.10489	2.2548	0.0326
Glycine, serine, and threonine metabolism	0.11395	2.172	0
Cysteine and methionine metabolism	0.13185	2.0261	0.12685
Pyrimidine metabolism	0.14068	1.9612	0.07132
Aminoacyl-tRNA biosynthesis	0.17315	1.7536	0
Purine metabolism	0.2087	1.5669	0.00969

[a] The pathway analysis was performed on MetaboAnalyst using the web-site built-in function.
[b] The *p* value was calculated from the pathway enrichment analysis.
[c] Impact was calculated from pathway topology analysis.
