# Supplementary material for: Machine learning of serum metabolic patterns encodes early-stage lung adenocarcinoma
Source: Nat Commun. 2020 Jul 16;11:3556. doi: 10.1038/s41467-020-17347-6 (PMC7366718; doi:10.1038/s41467-020-17347-6)
Supplement: Supplementary file 2 — Reporting summary [file 41467_2020_17347_MOESM2_ESM.pdf]

nature research

Corresponding author(s): Kun Qian

Last updated by author(s): Jun 5, 2020

Reporting Summary

Nature Research wishes to improve the reproducibility of the work that we publish. This form provides structure for consistency and transparency in reporting. For further information on Nature Research policies, see Authors & Referees and the Editorial Policy Checklist .

Statistics

For all statistical analyses, confirm that the following items are present in the figure legend, table legend, main text, or Methods section.

n/a	Confirmed	
☐	☒	The exact sample size (*n*) for each experimental group/condition, given as a discrete number and unit of measurement
☐	☒	A statement on whether measurements were taken from distinct samples or whether the same sample was measured repeatedly
☐	☒	The statistical test(s) used AND whether they are one- or two-sided<br>*Only common tests should be described solely by name; describe more complex techniques in the Methods section.*
☐	☒	A description of all covariates tested
☐	☒	A description of any assumptions or corrections, such as tests of normality and adjustment for multiple comparisons
☐	☒	A full description of the statistical parameters including central tendency (e.g. means) or other basic estimates (e.g. regression coefficient) AND variation (e.g. standard deviation) or associated estimates of uncertainty (e.g. confidence intervals)
☐	☒	For null hypothesis testing, the test statistic (e.g. *F*, *t*, *r*) with confidence intervals, effect sizes, degrees of freedom and *P* value noted<br>*Give P values as exact values whenever suitable.*
☒	☐	For Bayesian analysis, information on the choice of priors and Markov chain Monte Carlo settings
☒	☐	For hierarchical and complex designs, identification of the appropriate level for tests and full reporting of outcomes
☐	☒	Estimates of effect sizes (e.g. Cohen's *d*, Pearson's *r*), indicating how they were calculated

Our web collection on statistics for biologists contains articles on many of the points above.

Software and code

Policy information about availability of computer code

Data collection	DataExplorer (Version 4.5)
Data analysis	ORCA package (version 4.1.1)<br>SIMCA software package (version 14.0)<br>SPSS software (version 19.0)<br>MetaboAnalyst (online version 4.0, http://www.metaboanalyst.ca/)<br>MATLAB (version R2016a)

For manuscripts utilizing custom algorithms or software that are central to the research but not yet described in published literature, software must be made available to editors/reviewers. We strongly encourage code deposition in a community repository (e.g. GitHub). See the Nature Research guidelines for submitting code & software for further information.

Data

Policy information about availability of data

All manuscripts must include a data availability statement. This statement should provide the following information, where applicable:

- Accession codes, unique identifiers, or web links for publicly available datasets
- A list of figures that have associated raw data
- A description of any restrictions on data availability

The verification of the metabolites in this study was achieved by comparing the m/z features with human metabolome database (HMDB, http://www.hmdb.ca/). The data that support the findings of this study are available from the corresponding author upon reasonable request. The source data underlying Figs. 2b, c, d, 3, and 4b, f, and Supplementary Figs. 1, 2, 3, 4, 5, 6, 7, 9, 10, 11, 12, 13, 14, 15, 16, 17, 18, 19, 20, and 22 are provided as a Source Data file.

Field-specific reporting

Please select the one below that is the best fit for your research. If you are not sure, read the appropriate sections before making your selection.

- [x] Life sciences
- [ ] Behavioural & social sciences
- [ ] Ecological, evolutionary & environmental sciences

For a reference copy of the document with all sections, see nature.com/documents/nr-reporting-summary-flat.pdf

Life sciences study design

All studies must disclose on these points even when the disclosure is negative.

Sample size	We estimated the sufficient sample size with predicted power over 0.8 by power analysis using MetaboAnalyst (http://www.metaboanalyst.ca/).
Data exclusions	No data were excluded from our analyses.
Replication	We verified MS pattern reproducibility and 110 ± 3 out of 161 m/z features were normally distributed according to three control serum samples (each with 50 independent patterns). The independent replication was repeated 50 times for each sample.
Randomization	The participants were allocated randomly into training and validation sets by 5-fold cross-validation.
Blinding	The pathologists were blind to participants' clinical information and any other information about the acquisition results from MS analysis.

Reporting for specific materials, systems and methods

We require information from authors about some types of materials, experimental systems and methods used in many studies. Here, indicate whether each material, system or method listed is relevant to your study. If you are not sure if a list item applies to your research, read the appropriate section before selecting a response.

Materials & experimental systems

n/a	Involved in the study	
☒	☐	Antibodies
☒	☐	Eukaryotic cell lines
☒	☐	Palaeontology
☒	☐	Animals and other organisms
☐	☒	Human research participants
☒	☐	Clinical data

Methods

n/a	Involved in the study	
☒	☐	ChIP-seq
☒	☐	Flow cytometry
☒	☐	MRI-based neuroimaging

Human research participants

Policy information about studies involving human research participants

Population characteristics	A total of 481 subjects were consecutively recruited from 2014 to 2019 in Shanghai Chest Hospital, including 200 patients suffering early-stage LA (median age: 54; female/male: 100/100) and 200 healthy controls undergoing routine health care maintenance (median age: 53; female/male: 100/100) , 36 patients with squamous carcinoma (including squamous cell carcinoma and small cell carcinoma; median age: 54; female/male: 5/31), and 45 patients with benign lung diseases (including pneumonia, hamartoma, pulmonary tuberculosis, granuloma, and others; median age: 52; female/male: 25/20). No significant age difference was observed among all groups (F = 0.088, p = 0.767, by one-way analysis of variance (ANOVA)). Gender was also matched for healthy control and early-stage LA groups.
Recruitment	Subjects were consecutively recruited from 2014 to 2019 in Shanghai Chest Hospital. All patients were diagnosed by a panel of pathologists together and the tumours staged according to the international standards for tumour, node, and metastasis (TNM) staging of lung cancer. The pathologists were blind to any information about the acquisition results from MS analysis. Patients were excluded from the study if they had evidence of autoimmune syndromes or drugs. The potential self-selection bias may be the professional bias relying on the pathologists' experience and wrong labeling of recruited samples may lead to a useless classifier. The potential bias has been addressed by giving a unified diagnosis result from a panel of pathologists together.
Ethics oversight	All the investigation protocols in this study were approved by the institutional ethics committees of the Shanghai Chest Hospital and School of Biomedical Engineering, SJTU (KS1736).

Note that full information on the approval of the study protocol must also be provided in the manuscript.
